# Feeding a *Saccharomyces cerevisiae* fermentation product improves udder health and immune response to a *Streptococcus uberis* mastitis challenge in mid-lactation dairy cows

**DOI:** 10.1186/s40104-021-00560-8

**Published:** 2021-04-08

**Authors:** M. Vailati-Riboni, D. N. Coleman, V. Lopreiato, A. Alharthi, R. E. Bucktrout, E. Abdel-Hamied, I. Martinez-Cortes, Y. Liang, E. Trevisi, I. Yoon, J. J. Loor

**Affiliations:** 1 Department of Animal Sciences and Division of Nutritional Sciences, University of Illinois, Urbana, Urbana, IL 61801 USA; 2 Department of Animal Sciences, Food and Nutrition (DIANA), Università Cattolica del Sacro Cuore, 29122 Piacenza, Italy; 3 Department of Animal Production, College of Food and Agriculture Sciences, King Saud University, Riyadh, 11451 Saudi Arabia; 4 Department of Animal Medicine, Faculty of Veterinary Medicine, Beni-Suef University, Beni-Suef, 62511 Egypt; 5 Agricultural and Animal Production Department, UAM-Xochimilco, 04960 Mexico City, Mexico; 6 Diamond V, Cedar Rapids, IA USA

**Keywords:** Dairy cow, Mastitis, RNA-sequencing, *Saccharomyces cerevisiae* fermentation product, Udder health

## Abstract

**Background:**

We aimed to characterize the protective effects and the molecular mechanisms of action of a *Saccharomyces cerevisiae* fermentation product (NTK) in response to a mastitis challenge. Eighteen mid-lactation multiparous Holstein cows (*n* = 9/group) were fed the control diet (CON) or CON supplemented with 19 g/d NTK for 45 d (phase 1, P1) and then infected in the right rear quarter with 2500 CFU of *Streptococcus uberis* (phase 2, P2). After 36-h, mammary gland and liver biopsies were collected and antibiotic treatment started until the end of P2 (9 d post challenge). Cows were then followed until day 75 (phase 3, P3). Milk yield (MY) and dry matter intake (DMI) were recorded daily. Milk samples for somatic cell score were collected, and rectal and udder temperature, heart and respiration rate were recorded during the challenge period (P2) together with blood samples for metabolite and immune function analyses. Data were analyzed by phase using the PROC MIXED procedure in SAS. Biopsies were used for transcriptomic analysis via RNA-sequencing, followed by pathway analysis.

**Results:**

DMI and MY were not affected by diet in P1, but an interaction with time was recorded in P2 indicating a better recovery from the challenge in NTK compared with CON. NTK reduced rectal temperature, somatic cell score, and temperature of the infected quarter during the challenge. Transcriptome data supported these findings, as NTK supplementation upregulated mammary genes related to immune cell antibacterial function (e.g., *CATHL4*, *NOS2*), epithelial tissue protection (e.g. *IL17C*), and anti-inflammatory activity (e.g., *ATF3*, *BAG3*, *IER3*, *G-CSF*, *GRO1*, *ZFAND2A*). Pathway analysis indicated upregulation of tumor necrosis factor α, heat shock protein response, and p21 related pathways in the response to mastitis in NTK cows. Other pathways for detoxification and cytoprotection functions along with the tight junction pathway were also upregulated in NTK-fed cows.

**Conclusions:**

Overall, results highlighted molecular networks involved in the protective effect of NTK prophylactic supplementation on udder health during a subclinical mastitic event.

**Supplementary Information:**

The online version contains supplementary material available at 10.1186/s40104-021-00560-8.

## Background

Bovine mastitis, an inflammation of the mammary gland, is one of the most-common and economically-important diseases in the dairy industry. Primarily caused by bacterial intramammary infection (IMI), predominantly *Escherichia coli*, *Streptococcus uberis*, and *Staphylococcus aureus* [1], clinical cases affect almost the totality of producers (99.7%), and approximately a quarter of all cows in the United States [2]. Despite advances in treatment and prevention, these numbers have increased throughout the past 25 years [2–4]. With yearly prevention costs ranging between $70 to $100 per cow [5, 6], and a variable estimated cost to an American farmer of up to $420–450 per case [5, 7, 8], the economic impact of this disease is evident.

Traditionally, treatment of clinical cases of mastitis and control of mastitis in dairy herds utilize antimicrobial agents as a blanket approach of antibiotic. However, given societal concerns, dairy production systems have a renewed focus to reduce the reliance on nonselective antibiotic use and develop new and sustainable strategies to manage and control infectious disease [1]. Nutritional interventions emphasizing the immune system and prevention, rather than curing IMI, are of interest to researchers, feed companies, and producers. Fermentation products specifically derived from *Saccharomyces cerevisiae* (SCFP) were reported to increase leukocyte function both *in vitro* and *in vivo* [9, 10], and generated beneficial health effects in other physiological scenarios involving immunological challenges [11–14]. When their efficacy against mastitis was tested in 25 large-scale commercial herds throughout the United States, SCFP reduced incidence of mastitis, and lowered their linear score when present [15]. The SCFP used in that and the current study is a dried product that contains multiple vitamins and antioxidants, including polyphenols; however, the precise components and composition are proprietary and not publicly-available. In addition to antioxidants and vitamins found in SCFP, other bioactive compounds including fermentation end-products, β-glucans, and other components of the yeast cell can modulate the immune response in humans as well as animals by priming the innate and adaptive immune response through activation of immune cells [16, 17].

In the current study, our objective was to investigate the efficacy of SCFP (NutriTek®, Diamond V, Cedar Rapids, IA, USA) during a case of subclinical mastitis, modeled via an IMI challenge involving *Strep. uberis*, and to address its molecular mechanism of action. *Strep. uberis*, a Gram-positive and coagulase-negative bacteria, was chosen as one of the major pathogen that causes environmental mastitis, leading to both clinical and subclinical cases, some of which could turn chronic and eventually cow-associated [18].

Animal systemic response and the effect of SCFP supplementation on clinical parameters and a comprehensive blood biomarker panel were characterized. Furthermore, the local response and physiological mechanisms of mammary gland tissue were assessed via RNA-sequencing combined with bioinformatics analysis. Our hypothesis was that SCFP supplementation (bioactive mixture of compounds) would reduce the severity of mastitis by activating local leukocyte responses and epithelial tissue defenses.

## Methods

All procedures involving animals received approval from the Institutional Care and Use Committee at the University of Illinois, Urbana (protocol no. 17166).

### Experimental design and treatments

Eighteen multiparous Holstein cows past peak lactation (> 60 d postpartum) from the University of Illinois dairy herd were used for this study. To be eligible, cows must have exhibited composite milk somatic cell count < 200,000 cells/mL in two consecutive Dairy Herd Improvement Association (DHIA) samplings, and cows must not have been treated for clinical mastitis or any other diseases during early lactation. Furthermore, the current lactation must have not been the result of a twin pregnancy. Eligible cows were housed in individual tie-stalls, had free access to water, and were milked three times daily at 04:00, 12:00, and 19:30 h. Diet was provided ad libitum as a TMR once daily at 08:00 h. Ration was formulated to meet NRC (2001) requirements (Suppl. Table 1). After 1 week. of adaptation, cows were assigned to one of two treatments: a control group receiving the basal diet with no supplementation (CON; *n* = 9), and a supplemented group receiving 19 g/d of a *Saccharomyces cerevisiae* fermentation product (NTK; NutriTek®, Diamond V, Cedar Rapids, IA; *n* = 9) manually top-dressed over the basal diet, as per manufacturer recommendations. Groups were balanced for DIM, lactation number, intake, milk production, body weight, body condition score, and somatic cell count (Suppl. Table 2). Throughout the experiment cows were fed to ensure approximately 5 kg of daily refusals as fed, and feed offered was adjusted daily. Samples of the TMR, individual forages, cottonseed, concentrate mixes, and orts were collected weekly and analyzed for DM content (AOAC, 1995). As-fed formulations of TMR were adjusted weekly, if necessary, to account for changes in DM content of forages.

The experimental period lasted 75 days total and was divided into 3 phases: (i) phase 1, from the beginning of the experimental period until day 44, (ii) phase 2, from day 45 to 53, which represents the mastitis challenge period, and (iii) phase 3, from day 54 to 75, to observe the recovery from the challenge.

### Intramammary mastitis challenge

At day 45 of the experimental period, the first day of phase 2, all cows from both groups were subjected to a mastitis challenge via intramammary injection of 2500 colony forming units (CFU) of *Strep. uberis* (NIRD-0140 J strain; Drs. G. Dahl and C. Jeong, University of Florida, Gainesville, [19]). Upon delivery in agar, a bacterial colony was plated via sterile loop in sterile conditions on a tryptic soy agar (TSA) plate, and incubated overnight at 37 °C. The following day a single colony was selected and the process was repeated to ensure colony purity. Afterwards, purified colonies were inoculated into 1 mL of sterile 15% glycerol, vortexed thoroughly, and stored at − 80 °C. Two days prior the challenge, a piece of ice was retrieved from the stored vial, plated under sterile conditions on a TSA plate, and incubated at 37 °C overnight. The following day a single colony from the plate was inoculated into 5 mL of sterile tryptic soy broth (TSB) in a test tube and incubated in a shaking incubator at 37 °C overnight. The following day (morning of the challenge) bacterial concentration was calculated via optical density at 600 nm (OD_600_) measured by spectrophotometry (e.g., 1.0 OD_600_ equal 5 × 10^8^ CFU/mL). The bacterial solution was then serially diluted to a final concentration of 2500 CFU/mL in sterile PBS. Part of the inoculum was plated overnight at 37 °C on TSA to confirm the concentration. Resulting counts were between 2000 and 2500 CFU/mL.

After the morning milking, cows from both treatments (i.e., CON and NTK) were moved back to their stall for the *Strep. uberis* challenge. Before inoculation, the right rear quarter was cleaned with 70% isopropyl alcohol pads (Milk Check Teat wipes, Kleen Test Products Corp., Port Washington, WI, USA) and infused with 1 mL of bacterial inoculum with a sterile disposable syringe fitted with a plastic disposable teat cannula (Jorgensen Labs, Inc., Loveland, CO, USA). Immediately following inoculation, the inoculum was massaged upward in the quarter, and both rear teats were immersed in a teat disinfectant containing 1% iodine. Due to the labor intense sampling post inoculation, cows were inoculated in batches of 2 or 4, balanced per treatment. As indicated by visual and manual inspection and SCS data prior to inoculation, all inoculated quarters were healthy at the time of inoculation. After 36 h from the challenge, following the afternoon milking of the following day, both rear quarters were biopsied and treated with a daily infusion of 125 mg of ceftiofur HCl (SpectraMast LC, Pfizer Animal Health, Kalamazoo, MI, USA) for 7 consecutive days, terminating the challenge. Day 53 of the experimental period represents the end of phase 2, as the first day without antibiotic treatment. Animals were then followed (phase 3) to monitor their recovery from the mastitis event up to day 75 of the experimental period, which represent a full month (e.g., 30 d) from the inoculation intramammary of *Strep. uberis*. At the end of phase 2, two animals (one per each treatment group) developed severe mastitis in non-challenged quarters that sustained a reduction of DMI and near total cessation of lactation due to cross contamination of the infection to other quarters, and were removed from the study, reducing the number of animals per group to 8 in phase 3.

### Data and sample collection

#### Production parameters

Dry matter intake and milk production were recorded daily. Composite milk samples were prepared in proportion to milk yield at each milking, preserved (800 Broad Spectrum Microtabs II; D & F Control Systems Inc., San Ramon, CA, USA), and analyzed for contents of fat, protein, lactose, solid-not-fat (SNF), milk urea nitrogen (MUN), and SCC by mid-infrared procedures (AOAC International, 1995) in a commercial laboratory (Dairy Lab Services, Dubuque, IA, USA). Due to the consequences of the biopsy procedure on the challenged quarter (bloody milk and clots), daily production data from day 47 through 49 of the experiment were dropped from the final data set, and composite milk collection was not conducted during phase 2. On day 46 of the experimental period, production data from the evening was dropped as well as the first data after the surgical procedure. Body weight (BW) and body condition score (BCS) were recorded weekly. Three individual scorers were used at each time point and their scores were averaged to avoid bias.

#### Blood collection and analysis

Blood was sampled from the coccygeal vein at 0 (right after inoculation), 12, and 36 (right before biopsy) h from inoculation with *Strep. uberis*. Furthermore, samples were taken also at 3, 7, and 9 d after inoculation, which corresponded to 72, 168, and 216 h from the challenge. These samples were collected after the morning milking before feed distribution into vacutainer tubes containing lithium heparin (BD Vacutainer, BD and Co., Franklin Lakes, NJ, USA) and placed on ice. Plasma was obtained by centrifugation at 2000×*g* for 15 min at 4 °C and aliquots stored at − 80 °C until further analysis. Concentration of non-esterified fatty acids (NEFA), β-hydroxybutyrate (BHB), glucose, cholesterol, urea, minerals (Ca, P, Mg, Na, K, Cl, and Zn), ceruloplasmin, albumin, aspartate aminotransferase (AST), γ-glutamyl transferase (GGT), bilirubin, alkaline phosphatase, haptoglobin, creatinine, paraoxonase (PON), total reactive oxygen metabolites (tROM), myeloperoxidase (MPO), ferric reducing ability of plasma (FRAP), interleukin-1β (IL1β), interleukin-6 (IL6), and nitric oxides (NO_x_, NO_2_, and NO_3_) were analyzed as previously described, using a combination of ELISA, colorimetric, and UV methodologies [20, 21].

Samples collected at 0, 12, and 36 h from bacterial inoculation, including a baseline sample 48 h prior to inoculation, were also analyzed for innate immune cells response, measuring monocyte and neutrophil phagocytosis capacity and oxidative burst activity via a flow cytometry-based assay as previously described [21].

#### Clinical parameters

During phase 2, several health and clinical parameters were collected to assess the animal response to the intramammary challenge. Rectal body temperature, and heart and respiration rates were measured at − 24, 0, 6, 12, 18, 24, 30, and 36 h relative to *Strep. uberis* infusion. At the same time points udder temperature for each rear quarter was measured using an infrared camera (FLIR Systems, Inc., Wilsonville, OR, USA). Individual rear quarter milk samples were collected at − 1, 6, 13, 22, and 30 h approximately relative to *Strep. uberis* infusion (corresponding to five consecutive milking around the challenge time) for SCC analysis. SCC were converted to the linear somatic cell score (SCS), calculated as log_2_(SCC/100,000) + 3 where SCC is in units of cells/mL. For udder quarter data (temperature and SCS) a delta was calculated between the right rear (challenged) and left rear (non-challenged control) of each cow to better account for the individual response to the intramammary challenge (i.g., Δ = x_right_ − x_left_).

#### Liver and mammary gland biopsies

Liver and mammary gland rear quarter (challenged, and control) tissues were harvested approximately 36 h post intramammary challenge and on day 73 of the experimental period (~ 30 d post challenge) from all animals enrolled via percutaneous biopsy. The procedure was conducted under mild general anesthesia with xylazine and local anesthesia with lidocaine HCl. Due to the systemic nature of the liver as an organ, its tissue was sampled first. Liver biopsies were harvested using the same procedures as described previously [22]. For mammary gland tissue, a 3-cm incision was made through the skin and subcutaneous tissue, and then separated from the mammary capsule at the incision site. Approximately 150–200 mg of tissue was removed using a biopsy needle (Bard Magnum, 12 gauge × 16 cm; C. R. Bard Inc., Murray Hill, NJ, USA). Pressure was applied for 10 min to the incision area with sterile gauze to prevent excessive bleeding. The skin incision was then closed with 11 mm Michel clips and antimicrobial ointment applied to the incision site. Clots resulting from the biopsies were removed by hand at each milking over the following 3 d. Both tissue samples were immediately frozen in liquid nitrogen and stored at − 80 °C until further analysis.

### RNA-sequencing analysis

#### RNA extraction

Only the rear right quarter (challenged with the *Strep. uberis* inoculum) was used for mammary gland RNA extraction and subsequent analysis. Tissue was weighted (~ 0.05 g), immediately placed in QIAzol Lysis Reagent (Qiagen, Hilden, Germany) (1 mL) and homogenized using a Mini-Beadbeater-24 (Biospec Products Inc., Bartlesville, OK, USA) with two 30 s cycles, and 1 min incubation on ice in between the cycles. Samples were then centrifuged for 10 min at 12,000×*g* and 4 °C, and the supernatant was transferred to a separate tube and mixed with chloroform (0.2 mL). After centrifugation for 15 min at 12,000×*g* and 4 °C, the aqueous phase was transferred to a new tube, mixed with 100% ethanol (0.75 mL), and total RNA was cleaned using miRNeasy mini kit columns (Qiagen, Hilden, Germany) following manufacturer’s protocols. During purification, genomic DNA was removed using the RNase-Free DNase Set (Qiagen, Hilden, Germany). Quantity was determined using a Qubit Fluorometer (Invitrogen, Carlsbad, CA, USA), while integrity was assessed via a Fragment Analyzer™ (Agilent Technologies, Santa Clara, CA, USA). All samples had an RQN (RNA quality number) score greater than 7.0. RNA samples were stored at − 80 °C until analysis.

#### Library construction, and sequencing

A total of 16 animals (*n* = 8 per treatment group) were used for analysis. After quality control, samples were handed to the DNA Service Lab of the University of Illinois Roy J. Carver Biotechnology Center for library preparation and sequencing. RNA-seq libraries were constructed with the TruSeq Stranded mRNA Sample Prep kit (Illumina, San Diego, CA, USA). The final libraries were quantitated with a Qubit Fluorometer (Invitrogen, Carlsbad, CA, USA) and the average library fragment length was determined on a Fragment Analyzer™ (Agilent Technologies, Santa Clara, CA, USA). The libraries were diluted to 10 nM and further quantitated by qPCR on a CFX Connect Real-Time qPCR system (Biorad, Hercules, CA, USA) for accurate pooling of the barcoded libraries and maximization of number of clusters in the flowcell. Libraries were pooled by tissue and loaded on 4 lanes (2 per tissue) of an 8-lane flowcell for cluster formation and sequenced on an Illumina HiSeq 4000. The libraries were sequenced from one end of the cDNA fragments for a total of 100 bp. The fastq read files were generated and demultiplexed with the bcl2fastq v2.20 Conversion Software (Illumina, San Diego, CA, USA). The quality of the resulting fastq files was evaluated with the FastQC software, which generates reports with the quality scores, base composition, k-mer, GC and N contents, sequence duplication levels and overrepresented sequences. On average, 20–25 million reads per samples were obtained (Suppl. Table 3).

### Statistical analysis of phenotypic data

Statistical analysis was performed in SAS v9.4 (SAS Institute, Cary, NC, USA), and conducted by phase. All data were subjected to repeated measures ANOVA using PROC MIXED. Dietary treatment (TRT), time, and their interaction (TRT×time) were used as fixed effect. Time was omitted as a factor when only one data point was collected (e.g., in phase 2) for some measurements. Cow was the random effect. For analysis in phase 1, data collected during the adaptation phase, prior to treatment imposition, were used as a covariate. The Kenward-Roger statement was used to compute the degrees of freedom. Covariance structure for the repeated measurement were tested and the most appropriate (e.g., better fitting statistics) was chosen for each analysis. For intake, milk production and composition, BW, and BCS Autoregressive 1 was chosen. Spatial power was implemented for clinical data (body and udder temperature, respiration and hearth rate), blood analysis, and immune function data, while compound symmetry was selected for SCS data around the challenge. Normality of the residuals was checked via PROC UNIVARIATE. All data were normally distributed. Statistical significance was determined at *P* ≤ 0.05, whereas tendencies were considered at 0.05 < *P* ≤ 0.10.

### Transcriptome sequencing data processing and statistical analysis

Data were analyzed separately by tissue (mammary gland, or liver). Alignments and counts were performed on the Carl R. Woese Institute for Genomic Biology Biocluster of the University of Illinois High-Performance Biological Computing. Single-end reads were first filtered using Trimmomatic 0.33 [23] with a minimum quality score of 28 (i.e., base call accuracy of 99.84%) leading and trailing with a minimum length of 30 bp long and subsequently checked using FastQC 0.11.6 (Babraham Institute, Cambridge, UK). No reads were filtered as all had scores greater than 28. Reads were then mapped to the *Bos taurus* UMD 3.1.1 reference genome using default settings of STAR 2.6.0 [24]. Uniquely aligned reads were quantified using feature Counts [25] in the Subread package (v1.5.2) based on the Refseq gene annotation (% mapping reported in Suppl. Table 3).

Further data analysis was conducted using R. 3.5.1 (R Core Team, 2018). Reads uniquely assigned to a gene were used for subsequent analysis. Genes were filtered if 3 samples did not have > 1 count per million mapped reads. A TMM (trimmed mean of M-values) normalization was applied to all samples using edgeR [26]. After data were log_2_-transformed, edgeR was used to conduct differential expression analyses. The applied statistical model included dietary treatment, time, and their interaction as fixed effect. Differentially expressed genes (DEG) across time points were determined with a combination of fold-change (> 1.5 or < − 1.5) and raw *P*-value (< 0.05) thresholds to balance for reproducibility, sensitivity, and specificity or results [27, 28] as done in previous publications from our laboratory [29–31]. For the purpose of the current paper, bioinformatics analysis was conducted only on the treatment comparisons (NTK vs. CON) at 36 h post-challenge, to focus on the immediate molecular mechanism in response to the challenge, rather then the long-term recovery from it.

### Bioinformatics analysis

The dynamic impact approach (DIA) was used for Kyoto Encyclopedia of Genes and Genomes (KEGG) pathway analysis of DEG. The detailed methodology of DIA is described elsewhere [32]. Differently from the Excel-based published approach, the current analysis was performed in R 3.5.1. For the analyses, pathways with at least 4 genes represented in the transcriptome database were selected. Furthermore, pathways related to KEGG category “Human diseases” and Organismal system subcategories “Digestive system”, “Excretory system”, and “Sensory system” were not considered as not pertinent to the analyzed tissues. The DIA pathway analysis yields two core metrics for each pathway: impact and flux. The term impact refers to the biological importance of a given pathway as a function of the change in expression of genes composing the pathway (proportion of DEG and their magnitude) in response to a treatment, condition, or change in physiological state [32]. Consequently, the direction of the impact, or flux, characterizes the average change in expression as up-regulation/activation, down-regulation/inhibition, or no change.

## Results

### Performance

**Fig. 1:**
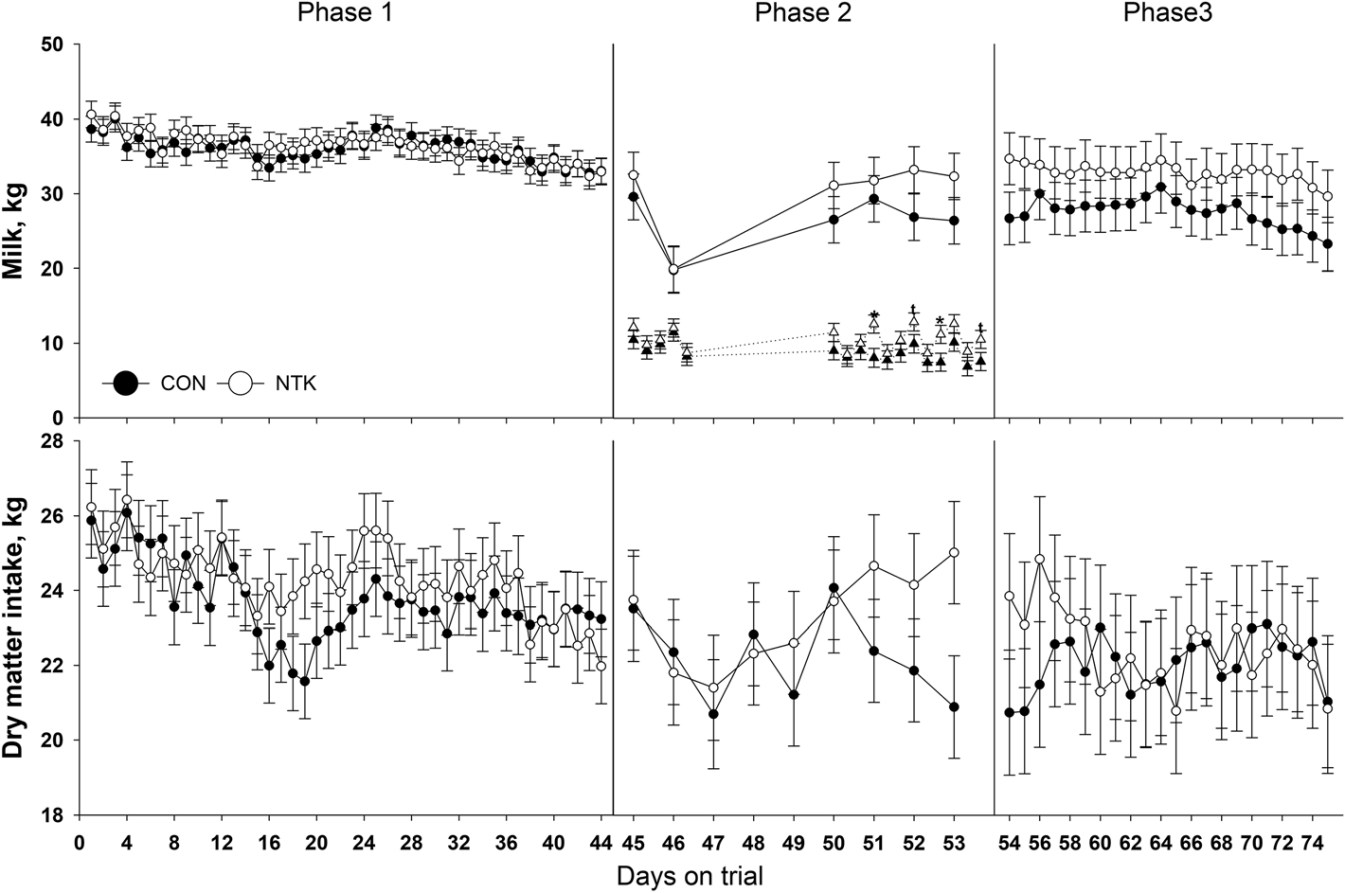
Milk yield and dry matter intake of cows supplemented with a *Saccharomyces cerevisiae* fermentation product (NTK) or fed a control diet (CON) subjected to an intramammary inflammation challenge with *Streptococcus uberis*. Data are separated according to experimental period: treatment feeding (Phase 1; 0–44 d), bacteria inoculation and challenge (Phase 2; 45–53 d), post antibiotic recovery (Phase 3; 54–75 d). For Phase 2, milk yield is represented both as daily values (sum of the three daily milkings, circles with full lines) or as single milking values (e.g., morning, noon, and evening yield for the corresponding day on trial, triangles with dotted lines). Superscripts represent differences among groups within each time point (*, *P* < 0.05; t, 0.05 < *P* < 0.10)

During phase 1, except for BCS (*P*_(TRT)_ = 0.07), no main effect of treatment was detected (*P*_(TRT)_ > 0.10). NTK cows tended to have slightly higher BCS (Table 1). Except for MUN (*P*_(Time)_ = 0.14), time effects were detected for all parameters (*P*_(Time)_ < 0.10). Milk yield, DMI (kg and %BW), fat and solids percentage, and SCS had a decreasing trend over time, while BW, BCS, and protein and lactose percentage tended to increase (Fig. 1, and Suppl. Figs. 1, 2, and 3). An interaction of treatment and time was observed for BW (*P*_(TRT×Time)_ = 0.02), but no statistical differences between treatment groups were detected at any time point.

**Table 1:** Milk yield and composition, dry matter intake, and body weight and body condition of cows supplemented with a *Saccharomyces cerevisiae* fermentation product (NTK) or a control diet (CON) subjected to an intramammary inflammation challenge with *Streptococcus uberis*. Data are separated according to experimental period: treatment feeding (Phase 1; 0–44 d), bacterial inoculation and challenge (Phase 2; 45–53 d), post antibiotic recovery (Phase 3; 54–75 d)

Parameter^**b**^	TRT^**a**^		SEM	***P***-value		
	CON	NTK		TRT	Time	TRT×Time
***Phase 1***						
Milk yield (daily), kg	35.98	36.33	1.22	0.84	<.0001	0.93
Milk yield (cumulative), kg	1571	1645	152	0.73	–	–
DMI, kg DM	23.73	24.30	0.63	0.53	0.001	0.95
DMI, % BW	1.44	4.46	0.04	0.69	0.001	0.98
BW, kg	754.3	759	4.7	0.49	<.0001	0.02
BCS	2.62	2.78	0.06	0.07	0.002	0.14
Fat, %	3.82	3.78	0.14	0.83	0.0002	0.35
Protein, %	3.69	3.72	0.03	0.54	0.01	0.15
Lactose, %	4.79	4.89	0.02	0.18	0.08	0.48
Solids, %	12.41	12.51	0.17	0.67	0.002	0.17
MUN, %	12.22	12.84	0.43	0.31	0.14	0.96
SCS	1.75	1.60	0.27	0.71	<.0001	0.45
***Phase 2***						
Milk yield (daily), kg	26.44	30.17	2.91	0.38	<.0001	0.08
Milk yield (by milking), kg	8.82	10.60	1.06	0.25	<.0001	0.01
Milk yield (cumulative), kg	160.9	196.5	19.7	0.22	–	–
DMI, kg DM	22.21	23.27	1.13	0.51	0.04	0.11
DMI, % BW	1.35	1.37	0.09	0.84	0.01	0.45
BW, kg	742.6	759.2	29.9	0.69	–	–
BCS	2.72	2.80	0.22	0.77	–	–
***Phase 3***						
Milk yield (daily), kg	27.54	32.84	3.35	0.28	0.0002	0.71
Milk yield (cumulative), kg	593.6	704.4	74.6	0.31	–	–
DMI, kg DM	22.04	22.47	1.50	0.85	0.46	0.11
DMI, % BW	1.31	1.32	0.10	0.96	0.50	0.08
BW, kg	766.6	773.7	16.8	0.77	0.89	0.95
BCS	2.75	2.92	0.15	0.43	0.74	0.15
Fat^c^, %	–	–	–	–	–	–
Protein, %	3.91	3.72	0.15	0.39	0.68	0.76
Lactose, %	4.71	4.75	0.03	0.35	0.49	0.08
Solids, %	11.97	11.49	0.38	0.39	0.44	0.95
MUN, %	10.35	10.24	1.13	0.94	0.05	0.91
SCS	2.86	2.65	0.52	0.78	0.006	0.74

^a^*TRT* Treatment, *CON* Control, *NTK* NutriTek® supplemented group (19 g/d)

^b^*DMI* Dry matter intake, *BW* Body weight, *BCS* Body condition score, *MUN* Milk urea nitrogen, *SCS* Somatic cell score, calculated as log_2_(SCC/100,000) + 3, were SCC (somatic cell count) was expressed in unit of cells per microliter

^c^Phase 3 fat content is not reported due to missing samples during shipping for analysis that reduced the n per group and prevented the model to converge

During phase 2, no main effect of treatment was detected for performance parameters (*P*_(TRT)_ > 0.10). The imposition of the challenge caused an initial depression of daily milk yield and DMI, with lowest values reached at day 46 and 47, respectively, followed by recovery of both parameters towards the end of the phase (*P*_(Time)_ < 0.05). (Table 1, Fig. 1, and Suppl. Fig. 1). Milk yield per milking displayed a daily fluctuation. An interaction of treatment and time was detected for both daily (*P*_(TRT×Time)_ = 0.08) and per milking (*P*_(TRT×Time)_ = 0.01) milk yield in phase 2. For daily milking no difference between groups was detected within each time point, while NTK cows had higher (*P* < 0.05) milk production in morning and night milkings on day 51 and 52, respectively, while there was a tendency (*P* < 0.10) for higher milk yield for NTK in the day 52 morning milking and day 53 evening milkings (Fig. 1).

Similar to phase 1 and 2, no main effect of treatment was detected during phase 3 (*P*_(TRT)_ > 0.10), while a time effect was observed for daily milk yield, percentage of MUN, and SCS (*P*_(Time)_ < 0.05), with all decreasing over time (Fig. 1, and Suppl. Fig. 3). An interaction of treatment and time was observed for lactose (*P*_(TRT×Time)_ = 0.08), as NTK supplemented cows had greater (*P* < 0.05) milk lactose percentage at 10 weeks. (Suppl. Fig. 3).

### Clinical parameters in response to bacterial inoculation

**Fig. 2:**
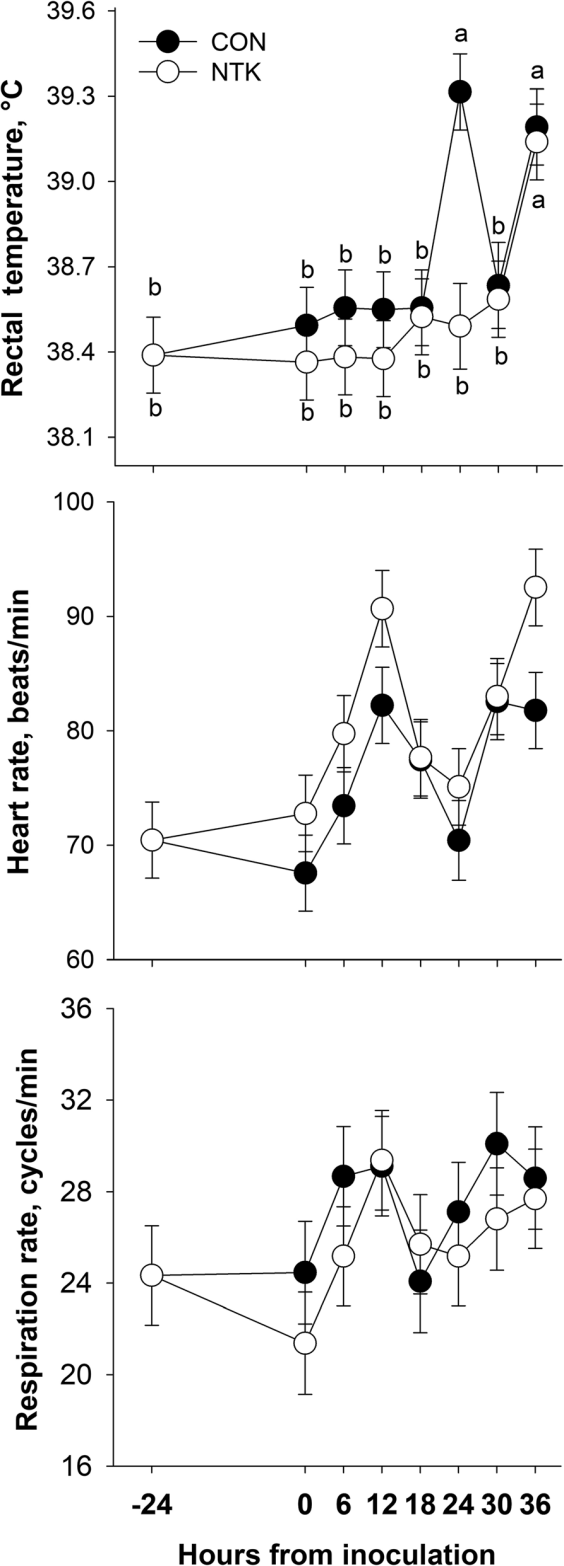
Clinical parameters of cows supplemented with a *Saccharomyces cerevisiae* fermentation product (NTK) or fed a control diet (CON) subjected to an intramammary inflammation challenge with *Streptococcus uberis* after 45 d of supplementation. Different superscripts indicate significant difference (*P* < 0.05) between values and are reported when the interaction of treatment and time is significant (*P* < 0.05)

**Fig. 3:**
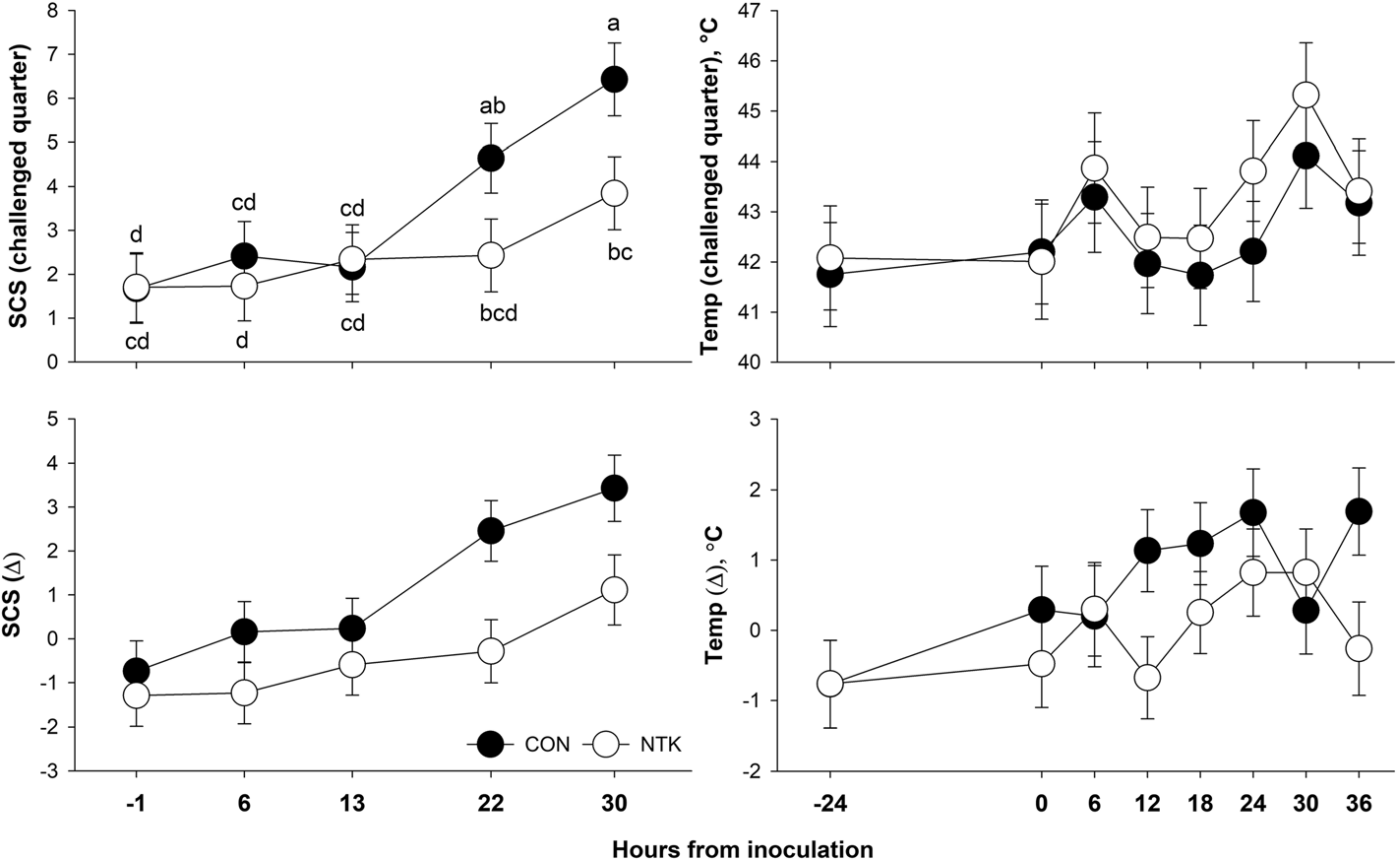
Linear somatic cell score and quarter temperature of cows supplemented with a *Saccharomyces cerevisiae* fermentation product (NTK) or fed a control diet (CON) subjected to an intramammary inflammation challenge with *Streptococcus uberis* after 45 d of supplementation. Data are expressed as recorded measurement in the challenged rear right quarter, or as delta subtracting the values measured in the non-challenged rear left quarter of each animal. Different superscripts indicate significant difference (*P* < 0.05) between values and are reported when the interaction of treatment and time is significant (*P* < 0.05)

**Fig. 4:**
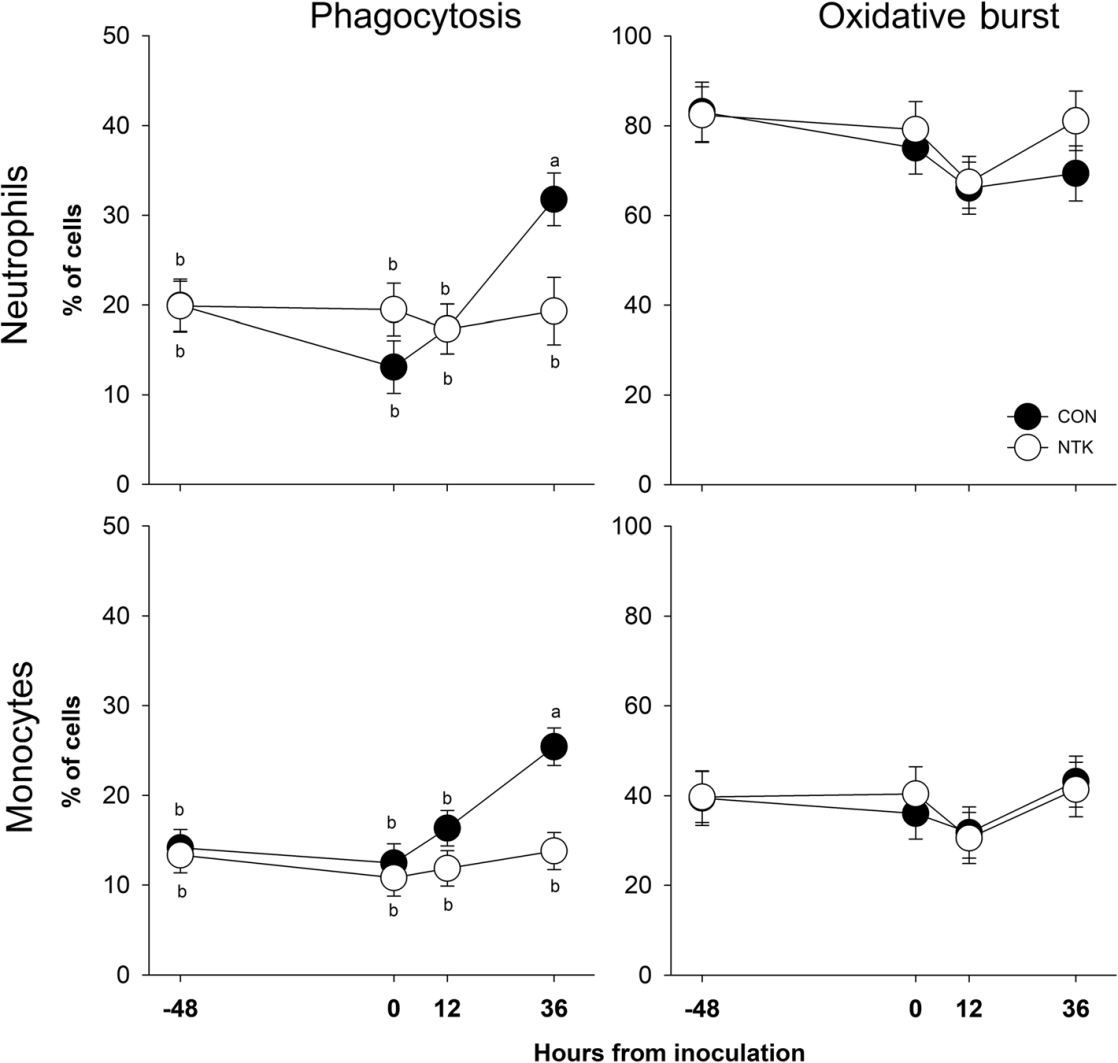
Circulating immune cells phagocytosis and oxidative burst capacity of cows supplemented with a *Saccharomyces cerevisiae* fermentation product (NTK) or fed a control diet (CON) subjected to an intramammary inflammation challenge with *Streptococcus uberis* after 45 d of supplementation. Different superscripts indicate significant difference (*P* < 0.05) between values and are reported when the interaction of treatment and time is significant (*P* < 0.05)

A time effect was observed for all parameters (*P*_(Time)_ ≤ 0.05) except for delta udder temperature (*P*_(Time)_ = 0.08), and monocyte oxidative burst (*P*_(Time)_ = 0.26, Table 2). Systemic clinical signs (rectal temperature, heart and respiration rate) displayed circadian changes with an overall upward trend in response to the challenge (Fig. 2). Similarly, udder clinical signs of inflammation (SCS, udder temperature, and their equivalent delta measurements) had an upward trend following the challenge (Fig. 3). Phagocytosis capacity of both monocytes and neutrophils had stable values from − 48 to 12 h relative to inoculation, with an elevated percentage at 36 h post-inoculation. However, neutrophil oxidative burst decreased from − 48 to 12 h relative to inoculation, followed by an increase to an intermediate level at 36 h (Fig. 4).

**Table 2:** Clinical parameters in response to an intramammary inflammatory challenge with *Streptococcus uberis* in animal fed a control diet (CON), or a diet supplemented with a *Saccharomyces cerevisiae* fermentation product (NTK)

Parameter^**2**^	TRT^**1**^		SEM	***P***-value		
	CON	NTK		TRT	Time	TRT×Time
**Phase 2**						
***Systemic***						
Rectal temp, °C	38.71^a^	38.53^b^	0.05	0.02	<.0001	0.05
Respiration rate, × min	75.73	80.24	1.93	0.11	<.0001	0.34
Heart rate, × min	27.05	25.7	1.69	0.58	0.0002	0.49
***Udder specific***						
Temp, challenged quarter, °C	42.55	43.18	0.71	0.54	0.005	0.96
Temp, delta, °C	0.717	0.002	0.251	0.06	0.08	0.37
SCS, challenge quarter	3.46	2.55	0.55	0.25	<.0001	0.29
SCS, delta	1.11^a^	-0.46^b^	0.52	0.05	<.0001	0.20
***Circulating immune function***						
Phagocytosis, monocytes, % cells	17.08^a^	12.45^b^	1.55	0.05	0.0005	0.01
Phagocytosis, neutrophils, % cells	20.54	19.00	1.84	0.55	0.02	0.03
Oxidative burst, monocytes, % cells	37.59	38.00	3.18	0.92	0.26	0.95
Oxidative burst, neutrophils, % cells	73.4	77.55	2.83	0.30	0.05	0.77

^1^*TRT* Treatment, *CON* Control, *NTK* NutriTek® supplemented group (19 g/d)

^2^Delta values are calculated as the difference between measures in the right rear quarter (challenged with 2500 CFU of *Strep. uberis*) and measures in the left rear quarter (unchallenged); *SCS* Somatic cell score, calculated as log_2_(SCC/100,000) + 3, were SCC (somatic cell count) was expressed in unit of cells per microliter

^a,b^Different superscripts denote statistical differences between the means (*P* < 0.05)

Dietary treatment had an effect on systemic and local clinical parameters and circulating immune function. Rectal temperature (Table 2, Fig. 2) was lower overall and specifically at 24 h post challenge for cows supplemented with NTK compared with control fed cows (*P*_(TRT)_ = 0.02 and *P*_(TRT×Time)_ = 0.05). At the udder, NTK supplemented cows tended to have a lower delta temperature (*P*_(TRT)_ = 0.06), and overall lower (*P*_(TRT)_ = 0.05) delta SCS (Table 2), mostly due to lower SCS values in the challenged quarter toward the end of the IMI challenge (Fig. 3). Feeding NTK lowered (*P*_(TRT)_ = 0.05) overall circulating phagocytosis capacity of monocytes (Table 2). An interaction with time (*P*_(TRT×Time)_ < 0.05) revealed how feeding NTK lowered both monocyte and neutrophil phagocytosis capacity at 36 h post inoculation (Fig. 4).

### Blood biomarkers

All metabolic parameters changed over time (*P*_(Time)_ < 0.05) with circadian concentration fluctuation in the first 36 h, and up or downwards trends post biopsy (Table 3, and Suppl. Fig. 4). A trend was observed for greater blood urea concentration in NTK supplemented animals, and an interaction of treatment and time was observed for creatinine concentration (*P*_(TRT×Time)_ = 0.05, Suppl. Fig. 4).

**Table 3:** Blood parameters in response to an intramammary inflammatory challenge with *Streptococcus uberis* in animal fed a control diet (CON), or a diet supplemented with a *Saccharomyces cerevisiae* fermentation product (NTK)

Parameter^**2**^	TRT^**1**^		SEM	***P***-value		
	CON	NTK		TRT	Time	TRT×Time
**Phase 2**						
***Metabolism***						
Glucose, mmol/L	4.68	4.74	0.12	0.70	<.0001	0.99
NEFA, mmol/L	0.119	0.124	0.013	0.81	0.04	0.16
BHB, mmol/L	0.513	0.519	0.031	0.87	<.0001	0.75
Cholesterol, mmol/L	5.65	5.19	0.32	0.32	0.0004	0.92
Urea, mmol/L	4.55	5.00	0.17	0.08	<.0001	0.40
Creatinine, μmol/L	79.33	80.05	1.91	0.79	<.0001	0.05
***Inflammation and acute phase proteins***						
IL-1β, pg/mL	80.93	47.44	24.7	0.35	0.13	0.05
IL-6, pg/mL	284.7	293.3	44.2	0.89	0.16	0.76
MPO, U/L	433.8	427.4	18.5	0.81	<.0001	0.27
Haptoglobin, g/L	0.555	0.546	0.101	0.94	<.0001	0.33
Albumins, g/L	37.84	37.93	0.52	0.90	<.0001	0.85
Bilirubin, total, μmol/L	2.38	2.12	0.13	0.17	0.06	0.11
Ceruloplasmin, μmol/L	3.18	3.14	0.24	0.90	<.0001	0.79
***Oxidative status***						
ROMt, mg H_2_O_2_/100 mL	16.57	16.26	0.86	0.80	<.0001	0.68
NO_x,_ μmol/L	21.91	21.55	0.50	0.62	0.01	0.93
NO_2,_ μmol/L	5.33	5.31	0.29	0.96	0.04	0.74
NO_3,_ μmol/L	16.72	16.15	0.36	0.28	0.15	0.97
FRAP, μmol/L	132.3	134.2	5.3	0.80	<.0001	0.35
Paraoxonase, U/mL	91.1	90.09	4.74	0.88	0.001	0.95
***Liver enzymes***						
Alkaline phosphatase, U/L	56.80	63.89	4.53	0.28	<.0001	0.54
AST, U/L	88.85	115.19	10.84	0.10	<.0001	0.40
GGT, U/L	31.32	38.06	4.15	0.27	<.0001	0.89
***Minerals***						
Ca, mmol/L	2.43^a^	2.55^b^	0.03	0.01	0.009	0.28
Cl, mmol/L	101.8	101.5	0.6	0.75	<.0001	0.34
K, mmol/L	4.55	4.57	0.05	0.80	0.004	0.83
Mg, mmol/L	1.06	1.01	0.02	0.09	<.0001	0.71
Na, mmol/L	142.8	143.7	0.4	0.17	<.0001	0.05
P, mmol/L	1.97	1.94	0.08	0.78	0.10	0.70
Zn, μmol/L	9.84	11.20	1.06	0.37	<.0001	0.66

^1^*TRT* Treatment, *CON* Control, *NTK* NutriTek® supplemented group (19 g/d)

^2^*NEFA* Non-esterified fatty acids, *BHB* β-hydroxy butyrate, *MPO* Myeloperoxidase, *IL1β and IL6* Interleukin 1 beta and 6, *NO*_*x*_ Nitric oxides, *FRAP* Ferric reducing ability of plasma, *ROMt* Total reactive oxygen metabolites, *GGT* γ-glutamyl transferase, *AST* Aspartate aminotransferase

No main effect of treatment (*P*_(TRT)_ > 0.10) was observed for inflammation biomarkers and acute phase proteins. Concentrations of MPO, haptoglobin, albumin, and ceruloplasmin were affected by time (*P*_(Time)_ < 0.0001), with a downward trend for albumin and an upward trend for others (Suppl. Fig. 5). A tendency was observed for changes in bilirubin over time (*P*_(Time)_ = 0.06), as its concentration tended to increase 36 h post inoculation. An interaction of dietary supplementation and time was observed for IL-1β (*P*_(TRT×Time)_ = 0.05) (Suppl. Fig. 5).

No treatment effect, or interaction with time, was detected for biomarkers of oxidative stress (*P*_(TRT and TRT×Time)_ > 0.10). Except for NO_3_ concentration (*P*_(Time)_ = 0.15), there was a Time effect for other parameters in this category (*P*_(Time)_ < 0.05). ROM concentration was higher at 168 and 216 h post inoculation, while NO_x_, NO_2_, FRAP, and PON concentrations were at their lowest at the same time points (Suppl. Fig. 6). FRAP concentration also peaked at 12 h post inoculation, while that of PON dipped at the same time point (Suppl. Fig. 7).

Time affected all measured liver enzymes (*P*_(Time)_ < 0.0001), with downward trends over time (Suppl. Fig. 7). Except for a tendency (*P*_(TRT×Time)_ > 0.10) for greater AST in NTK supplemented cows (Table 3), no main effect of treatment or interactions with time were observed for these parameters.

Except for phosphorus (Table 3) with a tendency (*P*_(Time)_ = 0.10), concentrations of minerals were affected by time (*P*_(Time)_ < 0.01). Magnesium and zinc concentrations were lowest at 36 and 72 h post inoculation, then increased to pre-challenge levels (Suppl. Fig. 8). Likewise, concentration of potassium was lowest at 36 h post inoculation, then increased to values equivalent to previous time points. Concentrations of chlorine and sodium peaked at 12 and 36 h post inoculation. Afterwards, sodium concentration returned to basal levels, whereas chlorine was stable at intermediate values compared with 0, 12, and 36 h post inoculation. A main effect of dietary supplementation was observed for calcium concentration (*P*_(TRT)_ = 0.01), and an interaction with time was identified for sodium concentration (*P*_(TRT×Time)_ = 0.05). NTK cows displayed greater overall levels of calcium, and higher levels of sodium at 0 and 36 h post inoculation (Suppl. Figs. 5 and 8).

### RNA sequencing and bioinformatics analysis

Liver and mammary gland samples from the right rear quarter (inoculated with *Strep. uberis*) collected 36 h post inoculation and on day 73 of the experimental period were used for transcriptomic analysis. However, the current manuscript focuses specifically on the treatment comparison (NTK vs. CON) at 36 h post inoculation.

**Fig. 5:**
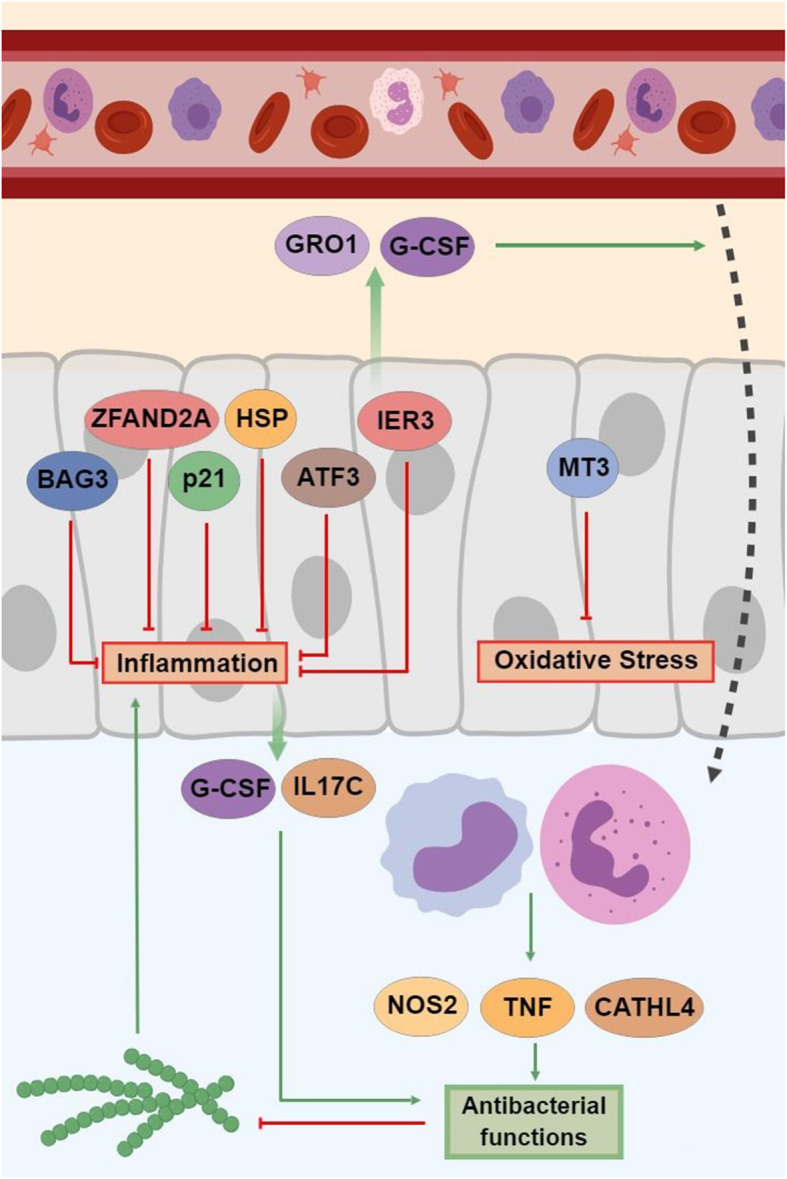
Molecular model summarizing the nutrigenomic effects on mammary gland tissue of dietary supplementation with a *Saccharomyces cerevisiae* fermentation product (NTK) compared to animals fed a control diet in response to an intramammary inflammatory challenge with *Streptococcus uberis*. From top to bottom the figure represents the mammary gland circulatory system, the mammary epithelium, and the lumen, where monocytes and neutrophils migrate in response to bacteria inoculation. The represented genes were upregulated by NTK supplementation, leading to the upregulation of functions in green, and downregulation of functions in red

**Fig. 6:**
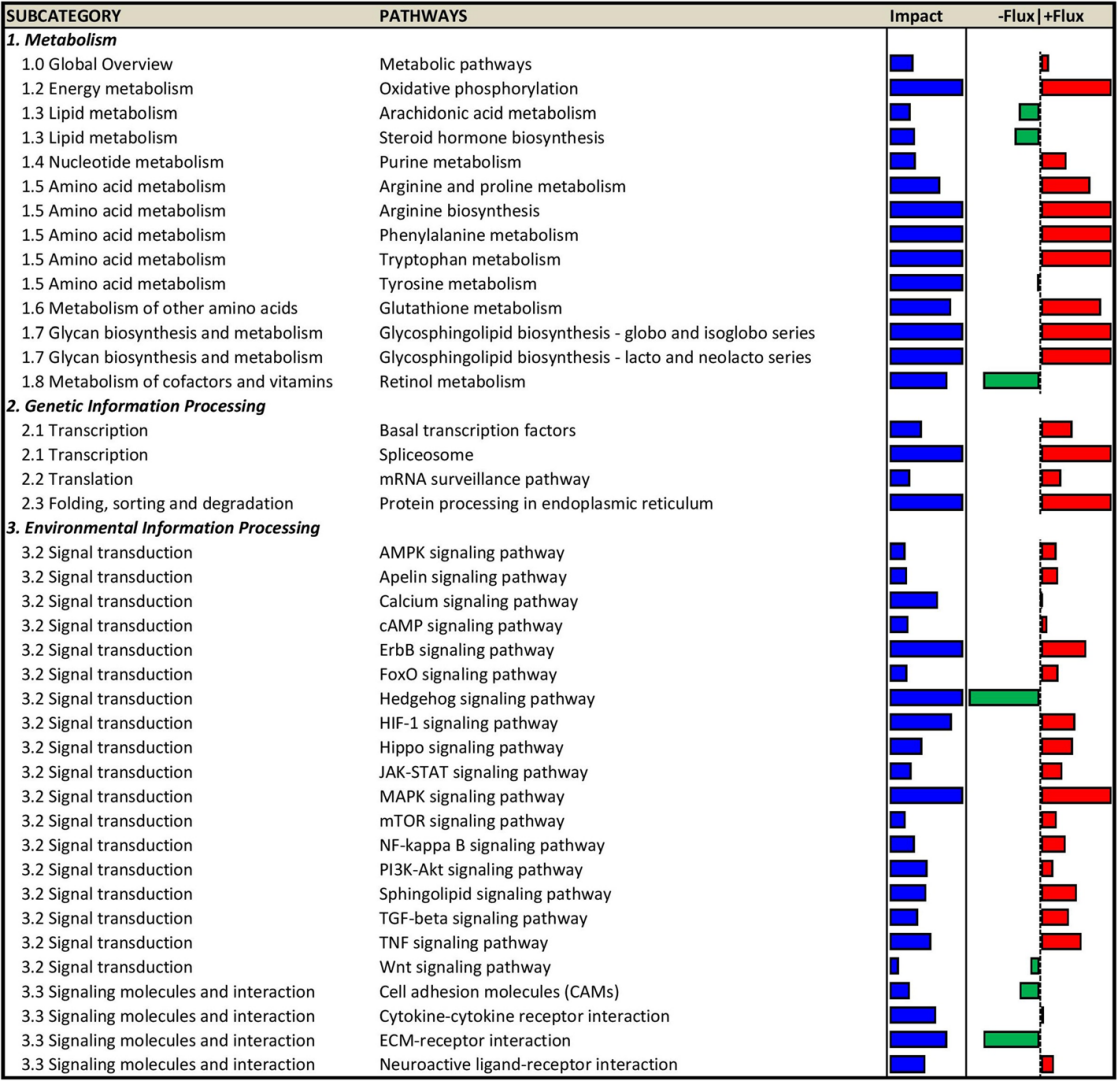
Activity of ‘metabolism’, ‘genetic information processing’, and ‘environmental information processing’ related pathway in mammary gland tissue collected 36 h post inoculation with *Streptococcus uberis* in dairy cows supplemented with a *Saccharomyces cerevisiae* fermentation product (NTK) compared to animals fed a control diet (CON). The impact (blue) represents the biological importance of the pathway, while the impact its direction of regulation. Positive flux (red) indicate upregulation, while negative flux (green) indicates downregulation of a pathway activity in the comparison NTK vs. CON

**Fig. 7:**
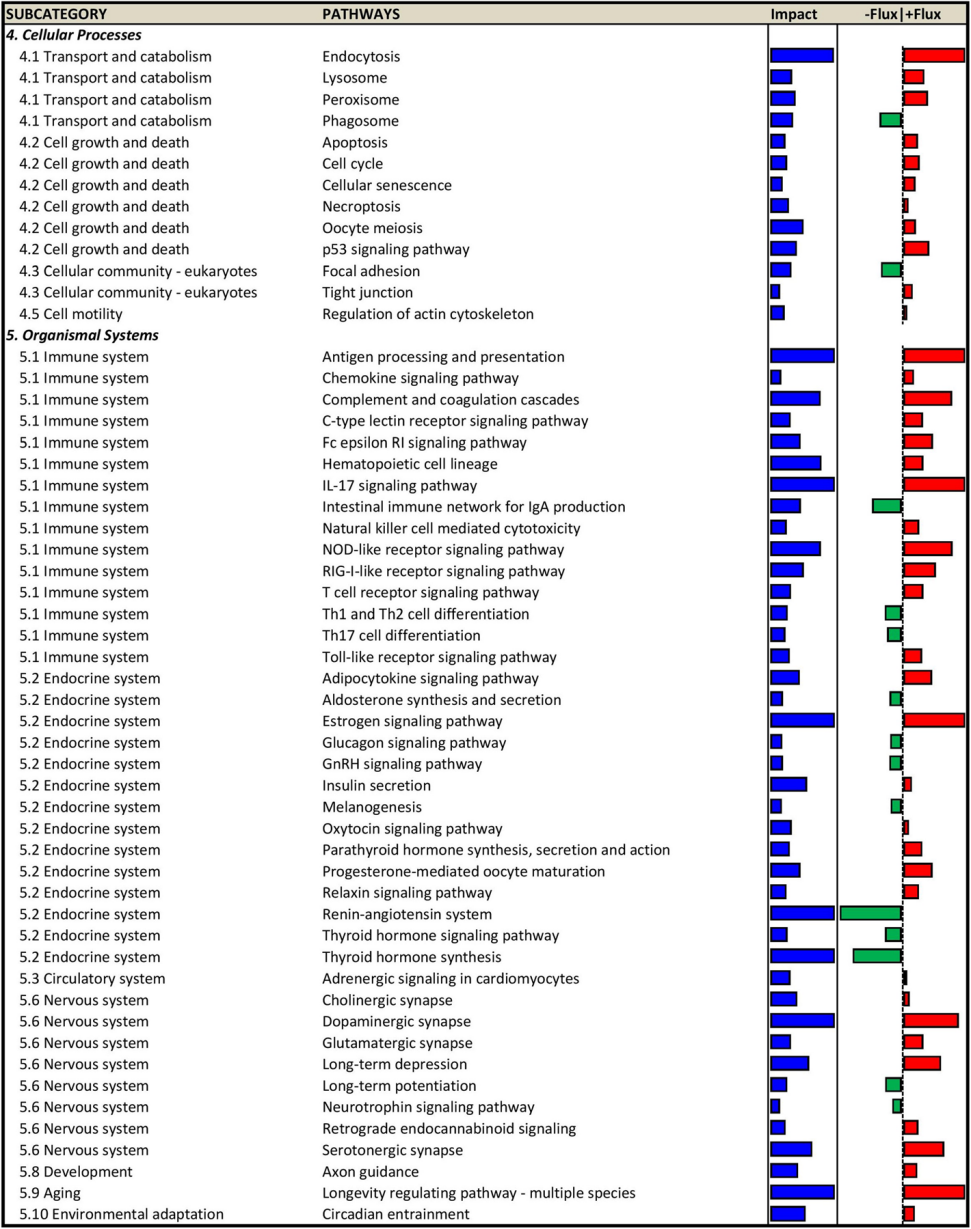
Activity of ‘cellular processes’, and ‘organismal systems’ related pathway in mammary gland tissue collected 36 h post inoculation with *Streptococcus uberis* in dairy cows supplemented with a *Saccharomyces cerevisiae* fermentation product (NTK) compared to animals fed a control diet (CON). The impact (blue) represents the biological importance of the pathway, while the impact its direction of regulation. Positive flux (red) indicate upregulation, while negative flux (green) indicates downregulation of a pathway activity in the comparison NTK vs. CON

In the mammary gland, NTK supplementation caused differential expression of 150 genes at 36 h post inoculation, of which 102 were upregulated and 42 downregulated (Suppl. Tables 4 and 5). NTK supplementation impacted a total of 94 pathways, of which 72 were upregulated and 22 downregulated. The summary of the DIA analysis (Suppl. Fig. 9) revealed an overall broad impact of NTK supplementation on non-metabolic pathways, with a general upregulation. Metabolically, NTK supplementation impacted pathways related to energy, lipid, and amino acids metabolism, while no effect on carbohydrate and secondary metabolism was detected. A model was built using the most recurring genes in the impacted metabolites (Fig. 5). All impacted pathways in the mammary gland are reported in Figs. 6 and 7.

At the level of the liver, NTK supplementation caused the differential expression of 50 genes at 36 h post inoculation, of which 28 were upregulated and 22 were downregulated (Suppl. Table 6). Pathway analysis revealed fewer changes in this tissue compared with mammary gland (Suppl. Fig. 9). NTK supplementation affected a total of 56 pathways, of which 43 were upregulated and 13 downregulated. Limited effects were observed metabolically, with only lipid and amino acid metabolism slightly impacted, and downregulated by NTK supplementation. The effect on non-metabolic pathways was similar to that observed in the mammary gland, with broad upregulation of most pathways in these categories. All impacted pathways in liver tissue are reported in Suppl. Fig. 10.

## Discussion

Although feeding SCFP can modulate the immune response resulting in a beneficial response to the herd in terms of lower mastitis incidence and a reduction in linear score [15], mechanisms of action remain unclear. The present study provides evidence of how NTK improves cow responses to an IMI challenge of *Strep. uberis* by increasing their bactericidal and functional capacity, while protecting the host cells from the inflammatory damage and cytotoxic side effects of an innate immune response.

After 45 d of supplementation, i.e. at the end of phase 1, no performance differences were detected between groups. A published meta-analysis observed increased production performance in dairy cows fed SCFP, with greater intakes in early-lactation and the reverse in late-lactation resulting in overall greater efficiency [33]. The lack of change in production parameters in the current study is probably attributable to lack of sufficient biological replication. In fact, when a larger cohort of dairy cows at a similar stage of lactation as in the present study was supplemented with the same SCFP, both milk production and efficiency improved [34]. Regarding udder health, no effect of NTK was detected in phase 1, as SCS did not differ from CON. Previous reported effects were detected at the herd level [15], i.e. including cows at all stages of lactation, suggesting that the observed effects on mastitis incidence and linear score were driven by animals undergoing transition or drying off, when the incidence of the disease is most prevalent [35]. This suggestion is supported by the lack of changes in SCC in a study with mid-lactation dairy cows fed the same SCFP [34].

The occurrence of clinical signs (rectal temperature, heart and respiration rate, udder temperature, and SCS) indicated effectiveness of the pathogen in creating a subclinical mastitic event, an outcome frequently observed in the field during *Strep. uberis* IMIs [18]. This challenge remained confined to the mammary gland and did not become systemic, as there were no peripheral changes in blood acute-phase proteins (APP; albumin, haptoglobin), cytokines (IL1b, IL6), inflammatory markers (bilirubin, ceruloplasmin, liver enzymes, MPO), and biomarkers of oxidative stress (ROM, NO_x_, FRAP, PON). The lack of systemic changes could be due to the short duration of the challenge (36 h), while the systemic increase or decrease observed in these biomarkers beyond 36 h post inoculation were likely imputable to the surgical procedure (biopsy) of both mammary gland rear quarters. Despite the lack of changes in the first phase, when animals were kept under observation without the addition of external insults, effects of NTK supplementation were observed once the IMI challenge with *Strep. uberis* was imposed. The overall lower rectal temperature and SCS, together with lower corrected (infected minus control quarter) udder temperature and SCS underscored the effectiveness of NTK in reducing the severity of the IMI. In fact, by 30 h post inoculation, the infected quarter of NTK-supplemented cows had a SCS of 3.84, which corresponded to a SCC of 179,415 cells/mL, lower than the accepted threshold for a subclinical case [36], hence, classifying the quarter as healthy. In contrast, the infected quarter of control animals reached an SCS value of 6.43 (1,076,592 cells/mL), indicative of an ongoing mastitic event. These data validate what was previously observed in commercial operations, where cows fed the same SCFP experienced lower incidence of mastitis [15].

Once the validity of *Strep. uberis* IMI challenge as a mastitis model was established, and NTK supplementation counteracted its effects, the transcriptomic and bioinformatic analysis allowed a deeper understanding of the molecular mechanisms (at the tissue level) associated with the observed responses. A graphical model was built to summarize the information gathered via RNA-seq (Fig. 5). As an initial response to a pathogen invasion or injury, animal tissues send chemotactic signals into circulation to induce migration of leukocytes to the site of infection/lesion. Once they reach the site, leukocytes activate their antimicrobial machinery to fend off intruding pathogens. The four-fold upregulation of *GRO1* and *CSF3* (also known as *CXCL1* and *G-CSF*, respectively) in NTK supplemented animals might suggest a greater chemotaxis and influx of immune cells to the mammary gland [37, 38]. Lower SCS data in NTK animals suggest that is not the case. However, CSF3 was reported to reduce chemotaxis and increase phagocytosis and oxidative burst activity of human neutrophils [39, 40], suggesting its upregulation to be toward enhancing functionality of residing immune cells, rather than chemotactic functions. Additionally, the upregulation of *IL17C* in NTK supplemented cows supports the ability of SCFP to stimulate epithelial tissue production of antibacterial peptides and proinflammatory molecules enhancing host defense [41].

When evaluating effect of increased host bactericidal capacity, the transcriptomic analysis revealed upregulation of *TNF*, *CATHL4*, and *NOS2*. TNF is the master-regulator of the inflammatory response, which orchestrates a powerful antimicrobial response by a variety of mechanisms [42], while *CATHL4* (cathelicidin-4 or indolicidin) is a tridecapeptide amide found in bovine neutrophil cytoplasmic granules with potent bactericidal function, capable of virtual sterilization of a suspension of *S. aureus* and *E. coli* [43]. Its high tryptophan content, important for its function, could also explain the strong activation in metabolism of this amino acid in NTK-supplemented cows, as *CATHL4* was upregulated more than 20-fold in these animals.

The protein encoded by *NOS2* is responsible for the synthesis of nitric oxide (NO), a potent cytotoxic molecule produced by neutrophils with the ability to further modulate their functionality (e.g., neutrophils extracellular traps) [44]. Synthesis of NO depends on the cellular pool of arginine, which explains the activation by NTK supplementation of pathways such as ‘arginine biosynthesis’ and ‘arginine and proline metabolism’ (Fig. 6). Interestingly, *CATHL4* expression in the parenchymal tissue of lactating cows was observed to be reduced during mastitic events [45]. However, milk collected from cows undergoing a clinically-induced mastitic event had greater levels [46, 47]. As previous authors suggested [45], we believe that its increased expression in the current experiment arose from the presence of infiltrating immune cells, rather than the epithelial cells itself. As SCS were lower in NTK cows, compared with CON, the greater *CATHL4* and *NOS2* expression could indicate a stronger antimicrobial activity of the infiltrating immune cells in NTK-supplemented cows.

Bioinformatics analysis may have also detected two additional molecular signatures of a lower, or milder, mastitic event in the NTK-supplemented cows. The negative flux of ‘arachidonic acid metabolism’ suggested greater activation in control cows compared with NTK. This response was due to a combination of genes responsible for the synthesis of HETE (5- and 15-hydroxyeicosatetraenoic acids). Compared with healthy cows, a study involving dairy cows affected by coliform mastitis reported an elevation of these compounds in milk. Furthermore, the negative flux of ‘steroid hormone biosynthesis’ induced by greater expression of *STS* in control cows [48] suggested a poor pathogen resistance in control unsupplemented cows. When STS-deficient mice were infected with the fungus *Candida albicans*, they displayed a greater antifungal activity [49]. Thus, the lower expression of STS in NTK cows could have caused a greater resistance to the *Strep. uberis* IMI challenge. These two hypotheses match the observed clinical data, but need to be further tested *in vitro* and *in vivo* experiments.

The inflammatory response is a beneficial process with the goal of containing and eradicating threats to the host organism. However, both dysregulation of its magnitude or duration can lead to detrimental effects such as damage to the host tissue and its functionality, and multiple chronic pathologies [50]. Considering this, specialized compounds and pathways that protect from these effects while helping the resolution of inflammation gain strategic importance in restoring tissue homeostasis. As discussed above, the evidence indicates that NTK supplementation led to more efficient inflammatory responses to the mastitic challenge, thanks to increased antimicrobial and inflammatory functions of the innate immune system after its cells translocated to the infected tissue and were activated in situ. However, most of the arsenal deployed by these cells (e.g., free radicals, NO) also can have substantial cytotoxic effects on host cells. To counteract these effects and protect host cells, a series of molecular mechanisms were activated in the udder of NTK-supplemented cows. For instance, upregulation of ‘glutathione metabolism’ together with upregulation (23-fold) of *MT3* expression might have allowed the tissue to respond to the oxidative challenge imposed by the production of ROS from phagocytes [51, 52]. Activation of the ‘complement and coagulation cascades’ pathway, due to the increased expression of serpin-related genes, could have increased protection in NTK-supplemented cows from damage of cytotoxic enzymes leaked from the phagosome of immune cells [53].

Besides these mechanisms in response to specific stimuli, supplementation of NTK upregulated two more systems, the first of which was the heat shock protein (HSP) response, responsible for broader cytoprotection. HSPs, or stress proteins, are highly-conserved and present in all cells of all organisms [54]. They are a family of highly-homologous chaperone proteins induced in response to environmental, physical and chemical stresses and limit the consequences of damage while facilitating cellular recovery [54]. The coordinated activities of the HSPs modulate multiple events within apoptotic pathways to help sustain cell survival following damaging stimuli. These include, but are not limited to, the JNK, NF-κB and AKT cascades [55], all of which the bioinformatic analysis indicated were impacted by NTK supplementation. More recently, the HSP system was linked to the host immune response, as HSPs have been implicated in antigen presentation and cross-presentation [56]. In addition, extracellular HSPs can stimulate professional antigen-presenting cells of the immune system leading to the activation of macrophages and lymphocytes, and the activation and maturation of dendritic cells [54, 56]. Supplementation with NTK strongly impacted the HSPs response, upregulating many of its direct players during the mastitic challenge: *HSPA6* and *1A* (13.4 and 8.1 folds, respectively), *DNAJB13* and *DNAJB1* (4.4 and 3.3 folds, respectively), and *HSPH1* (2.9 folds). Additionally, NTK also upregulated genes indirectly involved [57, 58] with the HSPs response (*BAG3* and *ZFAND2A*, 2.5 and 2.6 folds, respectively).

The second system activated by NTK supplementation was the p21 cascade, responsible for the resolution of inflammation. Once inflammation is established and has accomplished its goal (e.g., induce a localized immune response to neutralize the invading pathogen), it must be resolved to restore tissue homeostasis and prevent long-term damage. Together with an increased activation of the ‘apoptosis’ pathway in NTK-fed cows already at 36 h post-inoculation, the upregulation of *CDKN1A* (or p21, 3.5 folds), which impacted and drove the positive flux of ‘p53 signaling pathway’ and ‘Cellular senescence’ pathways, suggested a resolution of the inflammation via immune cell apoptosis [59]. In fact, NTK cows never reached SCS high enough to be classified as having subclinical mastitis. Additionally, upregulation of *ATF3* (2.9 folds) and *IER3* (2.2 folds) further supported the idea of a resolving inflammatory response. *ATF3* is a transcription factor that controls inflammation by suppressing its signaling cascade to drive the tissue towards re-establishing homeostasis. In contrast, *IER3* is a regulatory gene activated in response to stressors which define subsets of regulatory networks in the elimination of pathogens, and in the restoration of epithelial barrier functions, while exerting effects as regulator of apoptosis [60]. Its deficiency has been linked with aberrant immune regulation and enhanced inflammation [60, 61].

These data taken together suggest a greater cytoprotective activity in the udder of NTK-supplemented cows. No histological analysis was undertaken in the current experiment. However, by using other data as proxy, we can speculate that NTK supplementation helped maintain integrity of the mammary gland epithelial tissue, preventing damage induced by the invading pathogen. In NTK cows, the bioinformatics analysis highlighted upregulation of the ‘tight junction’ pathway responsible for the synthesis and modulation of the cell-to-cell connection, maintaining an intact barrier as a first line of defense from *Strep. uberis* invasion. Furthermore, the higher calcium concentration in peripheral blood suggested a lower presence of pyrogen in NTK-supplemented cows. Calcium concentration, in fact, was shown to drop when LPS was infused via the jugular vein in cattle [62, 63], as it may be used to bind endotoxins in the process of clearing them from the circulation [64]. Lastly, phagocytosis activity of both neutrophils and monocytes increased in control animals at 30 h post-inoculation, while the value for NTK-supplemented animals remained constant. Because at the level of the mammary gland NTK-supplemented animals showed signs of increased immune cell activity, we argue the opposite effect (greater phagocytosis) observed in the circulation (where immune cells should travel in an inactive state) in control cows to be a sign of the presence of bacterial components leaked into circulation due to damage to the epithelial tissue in the udder (as suggested by bioinformatics and calcium data). This, then, led to the preemptive activation of cells in the circulation before extravasation to the infected mammary tissue. This hypothesis could not, however, be tested as these components (e.g., LTA in our case, as *S. uberis* is gram-positive) are quickly catabolized and not easy to detect in the circulation. An intact epithelium should lead to better recovery from the production drop induced by the mastitic event. Due to the limited number of animals, as previously discussed, our analysis was underpowered to effectively detect performance differences. However, towards the end of phase 2, few daily differences or tendencies were detected for greater milk production in NTK-supplemented cows. In phase 3, NTK-supplemented cows maintained numerically, but not statistically due to low number of cows and high standard errors, greater milk production compared with controls, returning to production values comparable to those pre-inoculation. Furthermore, despite the lack of statistical power and significance, a difference of 110 kg of cumulative milk production (or average of 5.3 kg/d/head) in phase 3 was observed for NTK animals.

As the IMI challenge did not cause major physiological or molecular changes systemically (e.g., no changes in APP, oxidative markers), we do not place much emphasis in the discussion of the limited transcriptional changes (50 total genes) observed at the hepatic level (pathway analysis available in Additional files). We hypothesize that the liver, as an immunologically-active organ, did not participate in the response to *Strep. uberis* inoculation. However, some of the transcriptomic changes may be indicative of a priming effect induced by NTK supplementation. For instance, the HSPs system was in fact not only activated in the mammary gland, but upregulation of its components was observed in the liver as well (9 total genes), with as high as 26-fold change in expression, and six common genes (*HSPA6*, *HASPA1A*, *HSPH1*, *DNAJB1, BAG3,* and *ZFAND2A*) were upregulated in both mammary gland and liver (Suppl. Table 7).

As part of the defense system, HSPs guarantee cell tolerance against a variety of stressors, and research on functional foods has revealed a number of substances likely to trigger cell protection through mechanisms that involve the induction of HSP expression in animals and humans studies [65]. As the inflammatory response in our experiment did not reach systemic levels and remained confined to the mammary gland, we hypothesize that these changes were induced constitutionally by the consumption of SCFP. Bioactive components identified in the literature include amino acids and polyphenols [65], all of which are present in the supplemented SCFP. The ability of SCFP activate the HSP response preemptively before a challenge is applied, as a priming effect of the animal capacity to respond to an insult, could be a major component of the protective effect of SCFP against a variety of external insults. Supplementation of SCFP, including the one used in the current experiment, has in fact displayed positive effects against thermal stress [66], mastitis [15], mycotoxins [14], and enteric bacteria [13], including physiological scenarios characterized by immunological challenges [11, 12].

We would like to remind the reader that production, metabolism, and gene expression profiles vary during lactation [67–69]. The reader is reminded to contextualize the reported results in reference to mid-lactation dairy cows despite our belief that the physiological mechanisms discussed should be maintained at all times during the production cycle, thus, as stated above, could be applied to different biological and environmental stressor. By mixing the product in herd TMR, use of this supplementation technology could be easily applied in the field. Thus, allowing for flexible dosing throughout the production cycle.

Future work should build upon these and other preliminary results [15] to assess the effect of SCFP on the capacity of the udder to clear bacteria after infection both in experimental and on-farm commercial scenarios. As such, relevant data to better characterize the mechanism of action of the product would be generated. Further, the conclusions below have been drawn from molecular changes observed in a cohort of 9 cows per group. In our experience (e.g. [31, 67, 69]), however, molecular changes are preserved and consistent enough such that 7–10 animals are sufficient to draw reliable and repeatable conclusions regarding physiological mechanisms.

## Conclusions

Supplementation with a commercially-available *Saccharomyces cerevisiae* fermentation product confirmed its potential in attenuating the severity of an experimentally-induced mastitic event with a common environmental pathogen (*Streptococcus uberis*). Molecular data generated highlight key features of the mechanisms of action of this product. Supplemented animals displayed a greater pathogen-killing capacity of such cells, and the activation of cellular mechanisms to enhance mammary gland cytoprotection against side effects of inflammation, while maintaining tissue integrity and health. Furthermore, similar effects were highlighted in the liver (e.g., priming of the heat shock protein response mechanism), which might explain the broad ameliorating effect of NTK supplementation against other environmental, immunological, and physiological stresses reported in the literature.

## Supplementary Information

**Additional file 1.** Provides detailed about diet composition (Suppl. Table 1), animal data prior to experiment (Suppl. Table 2), and complete results about the RNA-sequencing analysis, including sequencing and alignment performance (Suppl. Table 3), and full list of differentially expressed genes with relative fold-changes (Suppl. Tables 4, 5, and 6). Furthermore, it includes graphical representation of results reported only in tabular format in the main body: DMI as %BW (Suppl. Fig. 1), BW and BCS (Suppl. Fig. 2), milk composition (Suppl. Fig. 3), blood biomarkers of metabolic status, inflammation and APP, oxidative status, liver enzymes, and minerals (Suppl. Figs. 4, 5, 6, 7, and 8). Included are also the full results of liver DIA pathway analysis (Suppl. Fig. 10).

## Data Availability

The sequencing data have been submitted to Gene Expression Omnibus database and are available under the GEO Accession number GSE149194.

## Abbreviations

AKT: Protein kinase B
APP: Acute phase protein
AST: Aspartate aminotransferase
ATF3: Activating transcription factor 3
BAG3: BAG cochaperone 3
BCS: Body condition score
BHB: β-Hydroxybutyrate
BW: Body weight
CATHL4: Cathelicidin-4 or indolicidin
CDKN1A: Cyclin dependent kinase inhibitor 1A
CFU: Colony forming unit
CON: Control
CSF3: Colony stimulating factor 3
CXCL1: C-X-C motif chemokine ligand 1
DEG: Differentially expressed gene
DIA: Dynamic impact approach
DMI: Dry matter intake
DNAJB13: Dnaj heat shock protein family member B13
FRAP: Ferric reducing ability of plasma
G-CSF: Granulocyte colony stimulating factor
GGT: γ-Glutamyl transferase
GRO1: GRO1 oncogene
HETE: 5- and 15-Hydroxyeicosatetraenoic acids
HSP: Heat shock protein
IER3: Immediate early response 3
IL17C: Interleukin 17c
IL1b: Interleukin 1β
IL6: Interleukin 6
IMI: Intramammary infection
JNK: c-Jun N-terminal kinases
KEGG: Kyoto Encyclopedia of Genes and Genomes
MPO: Myeloperoxidase
MT3: Metallothionein 3
MUN: Milk urea nitrogen
MY: Milk yield
NEFA: Non-esterified fatty acids
NF-κB: Nuclear factor kappa beta
NO: Nitric oxide
NOS2: Nitric oxide synthase 2
NTK: NutriTek®
P1: Phase 1
P2: Phase 2
P3: Phase 3
PBS: Phosphate buffer saline
PON: Paraoxonase
SCC: Somatic cell count
SCFP: *Saccharomyces cerevisiae* fermentation product
SCS: Somatic cell score
SNF: Solid-not-fat
STS: Steroid sulfatase
TMM: Trimmed mean of M-values
TMR: Total mixed ration
TNF: Tumor necrosis factor α
tROM: Total reactive oxygen species
TSA: Tryptic soy agar
TSB: Tryptic soy broth
ZFAND2A: Zinc finger AN1-type containing 2A
