# Supplementary material for: Feeding a Saccharomyces cerevisiae fermentation product improves udder health and immune response to a Streptococcus uberis mastitis challenge in mid-lactation dairy cows
Source: J Anim Sci Biotechnol. 2021 Apr 8;12:62. doi: 10.1186/s40104-021-00560-8 (PMC8028142; doi:10.1186/s40104-021-00560-8)

**Supplemental Table 1.** Ingredient composition of the diet

| **Item** | **Content** |
| --- | --- |
| Ingredient, % of DM |  |
| Alfalfa hay | 9.26 |
| Ajipro-L-G3^1^ | 0.11 |
| Canola meal | 15.15 |
| Corn gluten meal | 4.56 |
| Corn silage | 41.33 |
| Ground shelled corn | 23.24 |
| ProVAAL2 AADvantage^2^ | 0.96 |
| Smartamine M^3^ | 0.07 |
| Soy hulls | 1.69 |
| Vitamin-mineral mix^4^ | 3.57 |
| Zinpro Availa-Dairy^5^ | 0.06 |
| Chemical analysis, % of DM |  |
| CP | 16.00 |
| ADF | 18.19 |
| NDF | 20.00 |
| NFC | 46.22 |
| Ether extract | 3.05 |
| NE_L_, Mcal/kg | 1.71 |
| ^1^Ajinomoto Heartland Inc. (Chicago, IL).  ^2^Perdue AgriBusiness (Salisbury, MD).  ^3^Adisseo (Alpharetta, GA).  ^4^Contained a minimum of 12.5% Ca, 10.4% Na, 2.2% Mg, 8.0% K, 0.1% S, 7.1% Se, 244.5 kIU of vitamin A/kg, 48.9 kIU of vitamin D_3_/kg, and 0.922 kIU of vitamin E/kg.  ^5^Zinpro Corporation (Eden Prairie, MN). | |

**Supplemental Table 2.** Initial parameters (average ±SD) at enrollment before treatment assignment.

| **Parameter^1^** | **CON** | **NTK** |
| --- | --- | --- |
| Lactation | 2.8 ± 0.8 | 3.1 ± 1.1 |
| DIM | 157 ± 80 | 156 ± 83 |
| Milk yield (daily), kg^2^ | 38.0 ± 12.2 | 39.8 ± 10.4 |
| DMI, kg DM^2^ | 24.5 ± 3.7 | 25.5 ± 4.4 |
| BW, kg^3^ | 745 ± 48 | 758 ± 58 |
| BCS^3^ | 2.60 ± 0.46 | 2.69 ± 0.53 |
| SCC, 10^3^ cells/mL^4^ | 63 ± 35 | 80.8 ± 39 |
| ^1^ CON: control; NTK: *Saccharomyces cerevisiae* fermentation product supplemented group; DIM: days in milk; DMI; dry matter intake; BW: body weight; BCS: body condition score; SCC: somatic cell count  ^2^ Average of data collected during the adaptation period (1 week before treatment allocation)  ^3^ One time measurement collected during the adaptation period (1 week before treatment allocation)  ^4^ Average of the two DHI tests conducted before enrollment | | |

**Supplemental Table 3.** RNA-sequencing and alignment performances conducted with STAR.

| **Sample** | **Tissue** | **Time** | **Treatment** | **Total reads** | **Uniquely mapped reads** | **% of uniquely mapped reads** |
| --- | --- | --- | --- | --- | --- | --- |
| 123_30D_R_TCATCCTT-AGCGAGCT_L00M_R1_001 | Mammary gland | 30 d | NTK | 27,365,531 | 25,622,705 | 93.63% |
| 123_36_R_GCCACAGG-CATGCCAT_L00M_R1_001 | Mammary gland | 36 h | NTK | 23,462,394 | 22,269,817 | 94.92% |
| 1416_30D_R_AGGTTATA-CAGTTCCG_L00M_R1_001 | Mammary gland | 30 d | NTK | 23,018,777 | 21,663,545 | 94.11% |
| 1416_36H_R_CTAGCGCT-GTGTAGAC_L00M_R1_001 | Mammary gland | 36 h | NTK | 26,371,130 | 25,504,335 | 96.71% |
| 609_30D_R_TACTCATA-CCTGTGGC_L00M_R1_001 | Mammary gland | 30 d | CON | 21,984,631 | 20,712,899 | 94.22% |
| 609_36_R_TGCGAGAC-CATTGTTG_L00M_R1_001 | Mammary gland | 36 h | CON | 26,463,728 | 25,260,468 | 95.45% |
| 8_30D_R_GTATGTTC-AACAGGAA_L00M_R1_001 | Mammary gland | 30 d | CON | 20,504,164 | 19,409,687 | 94.66% |
| 8_36_R_AGTACTCC-AACCTGTT_L00M_R1_001 | Mammary gland | 36 h | CON | 28,769,266 | 27,776,264 | 96.55% |
| 844_30D_R_TCTGTTGG-TCGAATGG_L00M_R1_001 | Mammary gland | 30 d | CON | 24,779,938 | 23,943,714 | 96.63% |
| 844_36_R_GTCTACAC-GCCAAGGT_L00M_R1_001 | Mammary gland | 36 h | CON | 26,676,700 | 25,480,021 | 95.51% |
| 8788_30D_R_CGTCTGCG-TTCACAAT_L00M_R1_001 | Mammary gland | 30 d | NTK | 20,725,722 | 19,868,107 | 95.86% |
| 8788_36_R_CATAGAGT-TGCCACCA_L00M_R1_001 | Mammary gland | 36 h | NTK | 29,238,686 | 28,274,012 | 96.70% |
| 89_30D_R_ACGCACCT-GGTGAAGG_L00M_R1_001 | Mammary gland | 30 d | NTK | 24,123,398 | 23,096,493 | 95.74% |
| 89_36_R_GACGTCTT-GGTTCACC_L00M_R1_001 | Mammary gland | 36 h | NTK | 26,908,669 | 25,877,631 | 96.17% |
| 8909_30D_R_GAACCGCG-TGACCTTA_L00M_R1_001 | Mammary gland | 30 d | CON | 27,071,711 | 25,375,030 | 93.73% |
| 8909_36_R_ATTGTGAA-TGCATTGC_L00M_R1_001 | Mammary gland | 36 h | CON | 22,442,047 | 21,149,841 | 94.24% |
| 8923_30D_R_CTGCTTCC-GATAGATC_L00M_R1_001 | Mammary gland | 30 d | CON | 22,439,186 | 20,950,007 | 93.36% |
| 8923_36_R_CCTTCACC-GACGCTCC_L00M_R1_001 | Mammary gland | 36 h | CON | 22,032,935 | 21,278,621 | 96.58% |
| 8950_30D_R_CGCTATGT-TCCGACAC_L00M_R1_001 | Mammary gland | 30 d | NTK | 21,117,013 | 19,743,027 | 93.49% |
| 8950_36_R_TGGCCGGT-TAGAGCGC_L00M_R1_001 | Mammary gland | 36 h | NTK | 27,855,988 | 26,403,294 | 94.78% |
| 9014_30D_R_TATCGCAC-CTTAGTGT_L00M_R1_001 | Mammary gland | 30 d | NTK | 22,923,945 | 21,909,760 | 95.58% |
| 9014_36_R_CAATTAAC-CGAGATAT_L00M_R1_001 | Mammary gland | 36 h | NTK | 24,419,341 | 23,745,994 | 97.24% |
| 9035_30D_R_GGTCACGA-GTATTATG_L00M_R1_001 | Mammary gland | 30 d | CON | 24,744,332 | 23,272,531 | 94.05% |
| 9035_36_R_TTCCTGTT-AGTATCTT_L00M_R1_001 | Mammary gland | 36 h | CON | 27,570,741 | 26,458,426 | 95.97% |
| 9227_30D_R_GTGAATAT-TCTCATTC_L00M_R1_001 | Mammary gland | 30 d | NTK | 27,120,691 | 25,420,581 | 93.73% |
| 9227_36_R_GTGTCGGA-GCTTGCGC_L00M_R1_001 | Mammary gland | 36 h | NTK | 21,813,367 | 20,414,318 | 93.59% |
| 9238_30D_R_AACTGTAG-ACGCCGCA_L00M_R1_001 | Mammary gland | 30 d | CON | 27,160,746 | 25,717,690 | 94.69% |
| 9238_36_R_ACACTAAG-ATCCATAT_L00M_R1_001 | Mammary gland | 36 h | CON | 24,265,746 | 23,711,179 | 97.71% |
| 9247_30D_R_TCGATATC-ACACGAGT_L00M_R1_001 | Mammary gland | 30 d | CON | 24,187,366 | 22,827,122 | 94.38% |
| 9247_36_R_ACAGGCGC-CTCTGCCT_L00M_R1_001 | Mammary gland | 36 h | CON | 25,623,830 | 25,035,661 | 97.70% |
| 9279_30D_R_CTCACCAA-CTAGGCAA_L00M_R1_001 | Mammary gland | 30 d | NTK | 22,039,959 | 20,661,075 | 93.74% |
| 9279_36_R_ACTCGTGT-ATTGGAAC_L00M_R1_001 | Mammary gland | 36 h | NTK | 28,390,730 | 27,363,463 | 96.38% |
| 123_30day_Liver_CATAGAGT-TGCCACCA_L00M_R1_001 | Liver | 30 d | NTK | 21,945,241 | 21,103,047 | 96.16% |
| 123_36h_Liver_CGGAACTG-CACTACGA_L00M_R1_001 | Liver | 36 h | NTK | 18,871,201 | 17,873,510 | 94.71% |
| 1416_30day_Liver_GTGAATAT-TCTCATTC_L00M_R1_001 | Liver | 30 d | NTK | 25,793,120 | 24,673,563 | 95.66% |
| 1416_36h_Liver_TAAGGTCA-TGTCGTAG_L00M_R1_001 | Liver | 36 h | NTK | 22,671,317 | 21,630,537 | 95.41% |
| 609_30day_Liver_TCTGTTGG-TCGAATGG_L00M_R1_001 | Liver | 30 d | CON | 21,982,292 | 20,929,626 | 95.21% |
| 609_36h_Liver_GTCTACAC-GCCAAGGT_L00M_R1_001 | Liver | 36 h | CON | 21,587,124 | 20,719,688 | 95.98% |
| 8_30day_Liver_TCATCCTT-AGCGAGCT_L00M_R1_001 | Liver | 30 d | CON | 20,314,707 | 19,248,993 | 94.75% |
| 8_36h_Liver_ATTGTGAA-TGCATTGC_L00M_R1_001 | Liver | 36 h | CON | 21,662,814 | 20,669,071 | 95.41% |
| 844_30day_Liver_GGTCACGA-GTATTATG_L00M_R1_001 | Liver | 30 d | CON | 20,261,046 | 19,473,569 | 96.11% |
| 844_36h_Liver_GTGTCGGA-GCTTGCGC_L00M_R1_001 | Liver | 36 h | CON | 17,773,263 | 16,878,568 | 94.97% |
| 8788_30day_Liver_TATCGCAC-CTTAGTGT_L00M_R1_001 | Liver | 30 d | NTK | 25,015,345 | 23,999,048 | 95.94% |
| 8788_36h_Liver_CAATTAAC-CGAGATAT_L00M_R1_001 | Liver | 36 h | NTK | 21,679,163 | 20,554,502 | 94.81% |
| 89_30day_Liver_CTCACCAA-CTAGGCAA_L00M_R1_001 | Liver | 30 d | NTK | 17,700,191 | 17,021,394 | 96.17% |
| 89_36h_Liver_ACTCGTGT-ATTGGAAC_L00M_R1_001 | Liver | 36 h | NTK | 22,402,050 | 21,565,713 | 96.27% |
| 8909_30day_Liver_AGGTTATA-CAGTTCCG_L00M_R1_001 | Liver | 30 d | CON | 19,200,778 | 18,279,770 | 95.20% |
| 8909_36h_Liver_CCATTCGA-GTTGTCCG_L00M_R1_001 | Liver | 36 h | CON | 20,721,235 | 19,793,001 | 95.52% |
| 8923_30day_Liver_ACAGGCGC-CTCTGCCT_L00M_R1_001 | Liver | 30 d | CON | 20,367,670 | 19,531,784 | 95.90% |
| 8923_36h_Liver_CCTTCACC-GACGCTCC_L00M_R1_001 | Liver | 36 h | CON | 21,404,145 | 20,600,347 | 96.24% |
| 8950_30day_Liver_GAACCGCG-TGACCTTA_L00M_R1_001 | Liver | 30 d | NTK | 28,372,000 | 27,153,922 | 95.71% |
| 8950_36h_Liver_GCCACAGG-CATGCCAT_L00M_R1_001 | Liver | 36 h | NTK | 19,818,762 | 18,914,874 | 95.44% |
| 9014_30day_Liver_CTGCTTCC-GATAGATC_L00M_R1_001 | Liver | 30 d | NTK | 17,915,560 | 17,194,662 | 95.98% |
| 9014_36h_Liver_TTCCTGTT-AGTATCTT_L00M_R1_001 | Liver | 36 h | NTK | 23,947,267 | 22,954,035 | 95.85% |
| 9035_30day_Liver_TGCGAGAC-CATTGTTG_L00M_R1_001 | Liver | 30 d | CON | 24,560,711 | 23,625,174 | 96.19% |
| 9035_36h_Liver_GATCTATC-ATGAGGCT_L00M_R1_001 | Liver | 36 h | CON | 19,409,837 | 18,637,619 | 96.02% |
| 9227_30day_Liver_AGTACTCC-AACCTGTT_L00M_R1_001 | Liver | 30 d | NTK | 22,514,479 | 21,558,508 | 95.75% |
| 9227_36h_Liver_CATAATAC-TTCTAACG_L00M_R1_001 | Liver | 36 h | NTK | 19,879,504 | 19,103,763 | 96.10% |
| 9238_30day_Liver_GACGTCTT-GGTTCACC_L00M_R1_001 | Liver | 30 d | CON | 23,811,507 | 22,976,227 | 96.49% |
| 9238_36h_Liver_TGCGGCGT-CCTCGGTA_L00M_R1_001 | Liver | 36 h | CON | 22,528,495 | 21,642,309 | 96.07% |
| 9247_30day_Liver_CGCTATGT-TCCGACAC_L00M_R1_001 | Liver | 30 d | CON | 24,657,478 | 23,744,605 | 96.30% |
| 9247_36h_Liver_TGGCCGGT-TAGAGCGC_L00M_R1_001 | Liver | 36 h | CON | 22,180,771 | 21,382,752 | 96.40% |
| 9279_30day_Liver_AACTGTAG-ACGCCGCA_L00M_R1_001 | Liver | 30 d | NTK | 24,698,732 | 23,603,348 | 95.57% |
| 9279_36h_Liver_ACACTAAG-ATCCATAT_L00M_R1_001 | Liver | 36 h | NTK | 19,895,092 | 18,897,898 | 94.99% |

**Supplemental Table 4.** Complete list of differentially expressed genes (Fold changes [FC]; NTK vs. CTR) in mammary gland biopsy collected 36 h post inoculation with *Streptococcus uberis* upregulated by the supplemented of a *Saccharomyces cerevisiae* fermentation product (NTK) compared to animals fed a control diet (CON).

| **EntrezID** | **Gene symbol** | **Gene name** | **FC** |
| --- | --- | --- | --- |
| 107131234 | *ITPRID1* | ITPR interacting domain containing 1 | 83.76 |
| 521163 | *ATP4A* | Atpase H^+^/K^+^ transporting subunit alpha | 44.82 |
| 520376 | *ELAVL3* | ELAV like RNA binding protein 3 | 34.37 |
| 515030 | *ACMSD* | Aminocarboxymuconate semialdehyde decarboxylase | 32.66 |
| 519580 | *HEPHL1* | Hephaestin like 1 | 24.36 |
| 613320 | *MT3* | Metallothionein 3 | 22.77 |
| 282166 | *CATHL4* | Cathelicidin 4 | 22.48 |
| 617538 | *IL17C* | Interleukin 17C | 16.73 |
| 100301161 | *KRT1* | Keratin 1 | 15.82 |
| 540452 | *SBSN* | Suprabasin | 13.95 |
| 539835 | *HSPA6* | Heat shock protein family A (Hsp70) member 6 | 13.42 |
| 280762 | *DDC* | Dopa decarboxylase | 11.67 |
| 107131141 | *LOC107131141* | Aromatic-L-amino-acid decarboxylase | 11.60 |
| 520603 | *KIAA0319* | Kiaa0319 | 11.23 |
| 100335759 | *SPINK6* | Serine peptidase inhibitor, Kazal type 6 | 8.22 |
| 282254 | *HSPA1A* | Heat shock protein family A (Hsp70) member 1A | 8.10 |
| 615277 | *LOC615277* | Acyl-coenzyme A thioesterase THEM4 | 7.33 |
| 504406 | *HYDIN* | HYDIN, axonemal central pair apparatus protein | 7.21 |
| 538679 | *LOC538679* | Ribonuclease, rnase A family, 7 | 6.88 |
| 789335 | *LOC789335* | Kelch-like protein 30 | 6.71 |
| 281347 | *NEFM* | Neurofilament medium | 6.59 |
| 100271926 | *CHST8* | Carbohydrate sulfotransferase 8 | 6.46 |
| 100295741 | *ZG16B* | Zymogen granule protein 16B | 6.18 |
| 505184 | *SERPINB2* | Serpin family B member 2 | 6.18 |
| 784932 | *LOC784932* | Serpin A3-7-like | 5.68 |
| 104975670 | *LOC104975670* | Uncharacterized LOC104975670 | 5.32 |
| 519403 | *ARC* | Activity regulated cytoskeleton associated protein | 5.25 |
| 104975109 | *LOC104975109* | Uncharacterized LOC104975109 | 5.21 |
| 538751 | *AREG* | Amphiregulin | 5.06 |
| 522712 | *VWA3A* | Von Willebrand factor A domain containing 3A | 4.99 |
| 100847618 | *LOC100847618* | Uncharacterized LOC100847618 | 4.86 |
| 104971754 | *LOC104971754* | Uncharacterized LOC104971754 | 4.76 |
| 100848466 | *PGR* | Progesterone receptor | 4.76 |
| 514873 | *ICAM4* | Intercellular adhesion molecule 4 | 4.74 |
| 782104 | *MYO15A* | Myosin XVA | 4.73 |
| 784163 | *AP3B2* | Adaptor related protein complex 3 subunit beta 2 | 4.63 |
| 104968898 | *LOC104968898* | Uncharacterized LOC104968898 | 4.56 |
| 615734 | *SLC5A8* | Solute carrier family 5 member 8 | 4.50 |
| 520270 | *DNAJB13* | Dnaj heat shock protein family (Hsp40) member B13 | 4.43 |
| 789175 | *LOC789175* | Beta-defensin 103B-like | 4.43 |
| 508869 | *RND1* | Rho family gtpase 1 | 4.22 |
| 281212 | *GRO1* | Chemokine (C-X-C motif) ligand 1 (melanoma growth stimulating activity, alpha) | 4.14 |
| 101903108 | *RNF212* | Ring finger protein 212 | 4.06 |
| 534626 | *GRIA3* | Glutamate ionotropic receptor AMPA type subunit 3 | 3.96 |
| 534439 | *PDZK1* | PDZ domain containing 1 | 3.92 |
| 282876 | *NOS2* | Nitric oxide synthase 2 | 3.90 |
| 281096 | *CSF3* | Colony stimulating factor 3 | 3.84 |
| 107132217 | *LOC107132217* | Uncharacterized LOC107132217 | 3.80 |
| 525013 | *TPO* | Thyroid peroxidase | 3.77 |
| 100847316 | *FAM177B* | Family with sequence similarity 177 member B | 3.77 |
| 505479 | *LOC505479* | Putative tetratricopeptide repeat protein 41 | 3.61 |
| 282244 | *GUCY2C* | Guanylate cyclase 2C | 3.57 |
| 505158 | *ICAM5* | Intercellular adhesion molecule 5 | 3.56 |
| 781657 | *C15H11orf91* | Chromosome 15 c11orf91 homolog | 3.55 |
| 513497 | *CDKN1A* | Cyclin dependent kinase inhibitor 1A | 3.46 |
| 280943 | *TNF* | Tumor necrosis factor | 3.39 |
| 617268 | *FAM131B* | Family with sequence similarity 131 member B | 3.35 |
| 617714 | *EFNB3* | Ephrin B3 | 3.32 |
| 615157 | *SCX* | Scleraxis bhlh transcription factor | 3.28 |
| 538426 | *DNAJB1* | Dnaj heat shock protein family (Hsp40) member B1 | 3.27 |
| 781188 | *MRAP2* | Melanocortin 2 receptor accessory protein 2 | 3.18 |
| 107133306 | *LOC107133306* | Uncharacterized LOC107133306 | 3.17 |
| 614873 | *B3GALT5* | Beta-1,3-galactosyltransferase 5 | 3.16 |
| 107133312 | *LOC107133312* | Ig lambda chain V-1 region-like | 3.11 |
| 531720 | *KLRG2* | Killer cell lectin like receptor G2 | 3.03 |
| 101902604 | *NAT8* | N-acetyltransferase 8 (putative) | 2.97 |
| 507165 | *HSPH1* | Heat shock protein family H (Hsp110) member 1 | 2.89 |
| 515266 | *ATF3* | Activating transcription factor 3 | 2.87 |
| 535207 | *CCDC65* | Coiled-coil domain containing 65 | 2.86 |
| 539093 | *GZMA* | Granzyme A | 2.84 |
| 617386 | *CFAP74* | Cilia and flagella associated protein 74 | 2.79 |
| 414350 | *PGA5* | Pepsinogen 5, group I (pepsinogen A) | 2.72 |
| 100848872 | *LOC100848872* | Uncharacterized LOC100848872 | 2.67 |
| 613974 | *ZFAND2A* | Zinc finger AN1-type containing 2A | 2.65 |
| 100847289 | *CCDC180* | Coiled-coil domain containing 180 | 2.65 |
| 782110 | *PPP2R2C* | Protein phosphatase 2 regulatory subunit Bgamma | 2.63 |
| 281685 | *CHRM3* | Cholinergic receptor muscarinic 3 | 2.60 |
| 538837 | *GPR141* | G protein-coupled receptor 141 | 2.52 |
| 782633 | *BAG3* | BCL2 associated athanogene 3 | 2.49 |
| 100848894 | *LOC100848894* | Uncharacterized LOC100848894 | 2.47 |
| 614460 | *PAG1* | Phosphoprotein membrane anchor with glycosphingolipid microdomains 1 | 2.46 |
| 338050 | *SERPINA5* | Serpin family A member 5 | 2.42 |
| 614840 | *GAS2* | Growth arrest specific 2 | 2.42 |
| 507061 | *DUSP5* | Dual specificity phosphatase 5 | 2.27 |
| 104970491 | *LOC104970491* | Uncharacterized LOC104970491 | 2.22 |
| 505455 | *IER3* | Immediate early response 3 | 2.22 |
| 523107 | *RASGEF1C* | Rasgef domain family member 1C | 2.19 |
| 101902845 | *LOC101902845* | Progesterone receptor-like | 2.19 |
| 107131980 | *LOC107131980* | Uncharacterized LOC107131980 | 2.18 |
| 519541 | *RNF125* | Ring finger protein 125 | 2.14 |
| 787419 | *C2CD4D* | C2 calcium dependent domain containing 4D | 2.12 |
| 617196 | *SLC26A7* | Solute carrier family 26 member 7 | 2.11 |
| 338078 | *STC1* | Stanniocalcin 1 | 2.11 |
| 538967 | *CDC42EP3* | CDC42 effector protein 3 | 2.11 |
| 617914 | *MAFF* | MAF bzip transcription factor F | 2.09 |
| 104973229 | *LOC104973229* | Uncharacterized LOC104973229 | 2.09 |
| 101906687 | *LOC101906687* | Uncharacterized LOC101906687 | 2.08 |
| 513231 | *GTF2B* | General transcription factor IIB | 2.08 |
| 617439 | *TNFRSF12A* | TNF receptor superfamily member 12A | 2.08 |
| 509227 | *FRK* | Fyn related Src family tyrosine kinase | 2.06 |
| 537051 | *ADAMTS9* | ADAM metallopeptidase with thrombospondin type 1 motif 9 | 2.05 |
| 516026 | *GPRC5A* | G protein-coupled receptor class C group 5 member A | 2.04 |

**Supplemental Table 5.** Complete list of differentially expressed genes (Fold changes [FC]; NTK vs. CTR) in mammary gland biopsy collected 36h post inoculation with *Streptococcus uberis* downregulated by the supplemented of a *Saccharomyces cerevisiae* fermentation product (NTK) compared to animals fed a control diet (CON).

| **EntrezID** | **Gene symbol** | **Gene name** | **FC** |
| --- | --- | --- | --- |
| 1.07E+08 | *LOC107132468* | Uncharacterized LOC107132468 | -50.62 |
| 281413 | *PNMT* | Phenylethanolamine N-methyltransferase | -22.65 |
| 1E+08 | *LRP2* | LDL receptor related protein 2 | -10.25 |
| 768025 | *KCNE2* | Potassium voltage-gated channel subfamily E regulatory subunit 2 | -9.69 |
| 1.02E+08 | *LOC101907544* | Tetratricopeptide repeat protein 9C-like | -9.27 |
| 1.05E+08 | *LOC104973891* | Uncharacterized LOC104973891 | -9.13 |
| 1.02E+08 | *LOC101904477* | ZW10 interactor pseudogene | -5.39 |
| 1.02E+08 | *LOC101902994* | Uncharacterized LOC101902994 | -5.34 |
| 539510 | *GDF10* | Growth differentiation factor 10 | -4.83 |
| 614062 | *CCDC60* | Coiled-coil domain containing 60 | -4.63 |
| 515917 | *LOC515917* | Pancreatic trypsin inhibitor | -4.36 |
| 1.02E+08 | *ANGPTL8* | Angiopoietin like 8 | -4.30 |
| 530102 | *COL6A6* | Collagen type VI alpha 6 chain | -3.99 |
| 1E+08 | *LOC100139075* | Retinol dehydrogenase 16-like | -3.90 |
| 281088 | *COMP* | Cartilage oligomeric matrix protein | -3.62 |
| 507016 | *CYP4F2* | Cytochrome P450, family 4, subfamily F, polypeptide 2 | -3.29 |
| 514492 | *TRIM6* | Tripartite motif containing 6 | -3.19 |
| 1.02E+08 | *LOC101903905* | Uncharacterized LOC101903905 | -3.13 |
| 540387 | *CYSLTR2* | Cysteinyl leukotriene receptor 2 | -3.12 |
| 528120 | *DOC2A* | Double C2 domain alpha | -3.04 |
| 493738 | *KLK1* | Kallikrein 1 | -3.00 |
| 282495 | *BOLA-DQB* | Major histocompatibility complex, class II, DQ beta | -2.97 |
| 512259 | *HPGD* | 15-Hydroxyprostaglandin dehydrogenase | -2.84 |
| 509206 | *STS* | Steroid sulfatase | -2.76 |
| 525636 | *RCAN2* | Regulator of calcineurin 2 | -2.71 |
| 1.01E+08 | *LOC100847613* | Uncharacterized LOC100847613 | -2.57 |
| 533449 | *ITGBL1* | Integrin subunit beta like 1 | -2.53 |
| 530719 | *CAMK2A* | Calcium/calmodulin dependent protein kinase II alpha | -2.46 |
| 534512 | *HMCN2* | Hemicentin 2 | -2.44 |
| 614455 | *RETSAT* | Retinol saturase | -2.41 |
| 541046 | *LGI2* | Leucine rich repeat LGI family member 2 | -2.40 |
| 281220 | *HAS2* | Hyaluronan synthase 2 | -2.39 |
| 1.07E+08 | *LOC107132767* | 60S Ribosomal protein L17 pseudogene | -2.39 |
| 533896 | *DUSP26* | Dual specificity phosphatase 26 | -2.32 |
| 281437 | *QPCT* | Glutaminyl-peptide cyclotransferase | -2.30 |
| 509235 | *ACE2* | Angiotensin I converting enzyme 2 | -2.27 |
| 615651 | *ZBED3* | Zinc finger BED-type containing 3 | -2.27 |
| 505371 | *TRAF3IP3* | TRAF3 interacting protein 3 | -2.27 |
| 617660 | *SYNDIG1L* | Synapse differentiation inducing 1 like | -2.21 |
| 281062 | *CDH2* | Cadherin 2 | -2.17 |
| 787068 | *INSRR* | Insulin receptor related receptor | -2.16 |
| 1E+08 | *BTNL9* | Butyrophilin like 9 | -2.14 |
| 1E+08 | *LOC100335990* | Small integral membrane protein 10-like protein 2A | -2.14 |
| 616916 | *REEP1* | Receptor accessory protein 1 | -2.09 |
| 505067 | *ZNF404* | Zinc finger protein 404 | -2.08 |
| 509585 | *ACKR3* | Atypical chemokine receptor 3 | -2.07 |
| 539528 | *KCNB1* | Potassium voltage-gated channel subfamily B member 1 | -2.06 |
| 537922 | *TNFRSF21* | TNF receptor superfamily member 21 | -2.03 |

**Supplemental Table 6.** Complete list of differentially expressed genes (Fold changes [FC]; NTK vs. CTR) in liver tissue collected 36h post mammary gland inoculation with *Streptococcus uberis* by the supplemented of a *Saccharomyces cerevisiae* fermentation product (NTK) compared to animals fed a control diet (CON).

| **EntrezID** | **Gene symbol** | **Gene name** | **FC** |
| --- | --- | --- | --- |
| 520376 | *ELAVL3* | ELAV like RNA binding protein 3 | 65.73 |
| 539835 | *HSPA6* | Heat shock protein family A (Hsp70) member 6 | 26.01 |
| 493725 | *DDX4* | DEAD-box helicase 4 | 14.50 |
| 1.02E+08 | *LOC100337081* | Adhesion G protein-coupled receptor E3 | 7.61 |
| 282254 | *HSPA1A* | Heat shock protein family A (Hsp70) member 1A | 6.64 |
| 540041 | *MATN3* | Matrilin 3 | 5.88 |
| 1.07E+08 | *LOC107131682* | Uncharacterized LOC107131682 | 4.62 |
| 1.07E+08 | *LOC107131843* | Insulin receptor substrate 1-like | 3.84 |
| 1.05E+08 | *LOC104974792* | Uncharacterized LOC104974792 | 3.68 |
| 507165 | *HSPH1* | Heat shock protein family H (Hsp110) member 1 | 3.65 |
| 536167 | *SH3TC2* | SH3 domain and tetratricopeptide repeats 2 | 3.19 |
| 782633 | *BAG3* | BCL2 associated athanogene 3 | 2.89 |
| 505165 | *RRAD* | RRAD, Ras related glycolysis inhibitor and calcium channel regulator | 2.82 |
| 1.01E+08 | *LOC100850437* | Uncharacterized LOC100850437 | 2.61 |
| 506096 | *HSPA4L* | Heat shock protein family A (Hsp70) member 4 like | 2.55 |
| 281885 | *KITLG* | KIT ligand | 2.53 |
| 533317 | *FLVCR1* | Feline leukemia virus subgroup C cellular receptor 1 | 2.50 |
| 538426 | *DNAJB1* | Dnaj heat shock protein family (Hsp40) member B1 | 2.36 |
| 613577 | *HERPUD1* | Homocysteine inducible ER protein with ubiquitin like domain 1 | 2.32 |
| 535327 | *MAPK13* | Mitogen-activated protein kinase 13 | 2.22 |
| 613974 | *ZFAND2A* | Zinc finger AN1-type containing 2A | 2.15 |
| 523322 | *ZNF667* | Zinc finger protein 667 | 2.14 |
| 541274 | *DNAJB4* | Dnaj heat shock protein family (Hsp40) member B4 | 2.14 |
| 510868 | *FAM19A3* | Family with sequence similarity 19 member A3, C-C motif chemokine like | 2.13 |
| 523303 | *NCR3LG1* | Natural killer cell cytotoxicity receptor 3 ligand 1 | 2.12 |
| 281831 | *HSPA8* | Heat shock protein family A (Hsp70) member 8 | 2.08 |
| 505972 | *LOC505972* | Fibroin heavy chain | 2.02 |
| 1.07E+08 | *FLVCR2* | Feline leukemia virus subgroup C cellular receptor family, member 2 | 2.01 |
| 1.07E+08 | *LOC107132288* | Uncharacterized LOC107132288 | -2.18 |
| 518597 | *MAB21L3* | Mab-21 like 3 | -2.18 |
| 523898 | *SULT1C2* | Sulfotransferase family, cytosolic, 1C, member 2 | -2.28 |
| 534591 | *KCNN4* | Potassium calcium-activated channel subfamily N member 4 | -2.44 |
| 404191 | *ANPEP* | Alanyl aminopeptidase, membrane | -2.45 |
| 532671 | *MCOLN2* | Mucolipin 2 | -2.47 |
| 787781 | *ARL5C* | ADP ribosylation factor like gtpase 5C | -2.61 |
| 1.05E+08 | *LOC104972879* | Uncharacterized LOC104972879 | -2.78 |
| 785997 | *LOC785997* | Olfactory receptor 4A47 | -3.01 |
| 1.02E+08 | *LOC101907446* | Uncharacterized LOC101907446 | -3.07 |
| 1E+08 | *LOC511354* | Immunoglobulin light chain variable region | -3.12 |
| 1.05E+08 | *LOC104976009* | Uncharacterized LOC104976009 | -3.12 |
| 282495 | *BOLA-DQB* | Major histocompatibility complex, class II, DQ beta | -3.47 |
| 1.02E+08 | *LOC101903139* | Uncharacterized LOC101903139 | -3.83 |
| 613562 | *SYT8* | Synaptotagmin 8 | -3.83 |
| 1E+08 | *LOC100140130* | Uncharacterized LOC100140130 | -4.01 |
| 404100 | *RAET1G* | Retinoic acid early transcript 1G | -4.12 |
| 615323 | *GRP* | Gastrin releasing peptide | -5.18 |
| 614096 | *ELOVL7* | ELOVL fatty acid elongase 7 | -6.25 |
| 510707 | *ULBP11* | UL16-binding protein 11 | -7.39 |
| 522099 | *GABRR2* | Gamma-aminobutyric acid type A receptor rho2 subunit | -12.70 |
| 1.02E+08 | *LOC101904573* | Spectrin alpha chain, non-erythrocytic 1 pseudogene | -19.60 |

**Supplemental Table 7**. List of genes differentially expressed simultaneously in liver and mammary gland tissue in response to supplementation with a *Saccharomyces cerevisiae* fermentation product (NTK) compared to animals fed a control diet (CON) 36 h post mammary gland inoculation with *Streptococcus uberis.*

|  |  |  | **Fold change** | | |
| --- | --- | --- | --- | --- | --- |
| **EntrezID** | **SYMBOL** | **Gene** | **Mammary Gland** | **Liver** |  |
| 520376 | *ELAVL3* | ELAV like RNA binding protein 3 | 34.37 | 65.73 |  |
| 539835 | *HSPA6* | Heat shock protein family A (Hsp70) member 6 | 13.42 | 26.01 |  |
| 282254 | *HSPA1A* | Heat shock protein family A (Hsp70) member 1A | 8.10 | 6.64 |  |
| 538426 | *DNAJB1* | Dnaj heat shock protein family (Hsp40) member B1 | 3.27 | 2.36 |  |
| 507165 | *HSPH1* | Heat shock protein family H (Hsp110) member 1 | 2.89 | 3.65 |  |
| 613974 | *ZFAND2A* | Zinc finger AN1-type containing 2A | 2.65 | 2.15 |  |
| 782633 | *BAG3* | BCL2 associated athanogene 3 | 2.49 | 2.89 |  |
| 282495 | *BOLA-DQB* | Major histocompatibility complex, class II, DQ beta | -2.97 | -3.47 |  |

**Supplemental Figure 1.** Dry matter intake as a percentage of body weight of cows supplemented with a *Saccharomyces cerevisiae* fermentation product (NTK) or fed a control diet (CON) subjected to an intramammary inflammation challenge with *Streptococcus uberis*. Data are separated according to experimental period: treatment feeding (Phase 1; 0-44 d), bacteria inoculation and challenge (Phase 2; 45-53 d), post antibiotic recovery (Phase 3; 54-75 d).

**
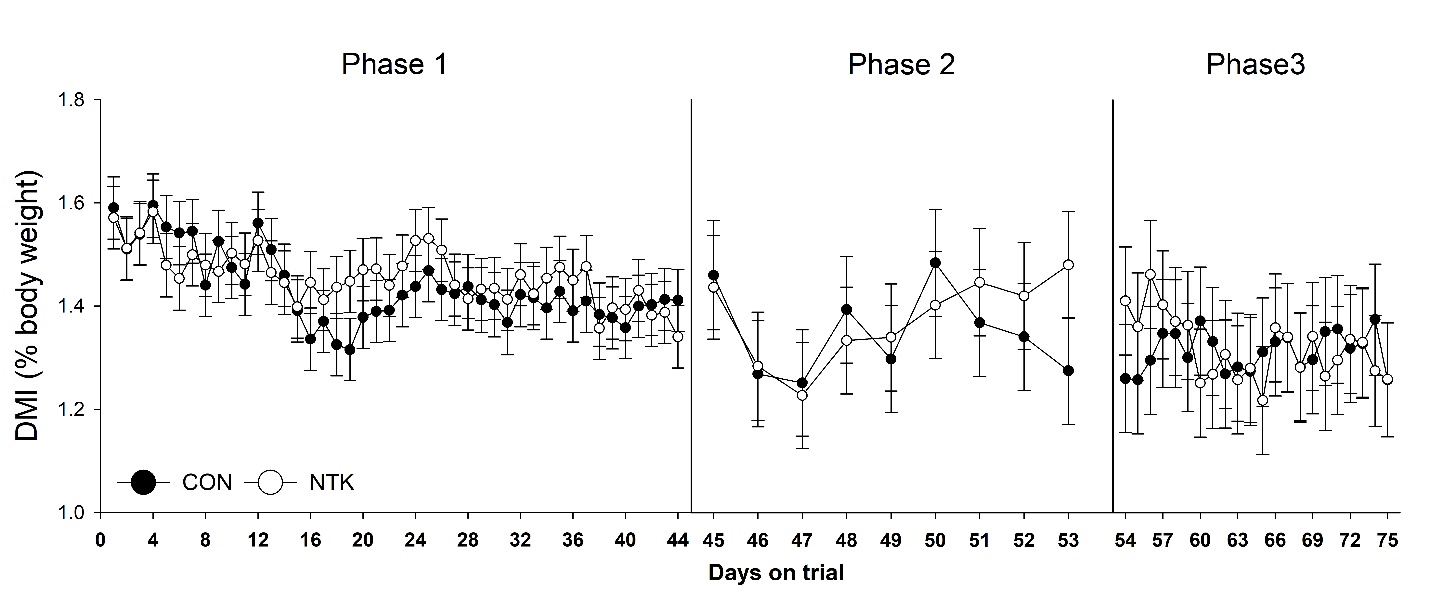
**

**Supplemental Figure 2.** Body weight and body condition score (BCS) of cows supplemented with a *Saccharomyces cerevisiae* fermentation product (NTK) or fed a control diet (CON) subjected to an intramammary inflammation challenge with *Streptococcus uberis*. Data are separated according to experimental period: treatment feeding (Phase 1; 0-44 d), bacteria inoculation and challenge (Phase 2; 45-53 d), post antibiotic recovery (Phase 3; 54-75 d).


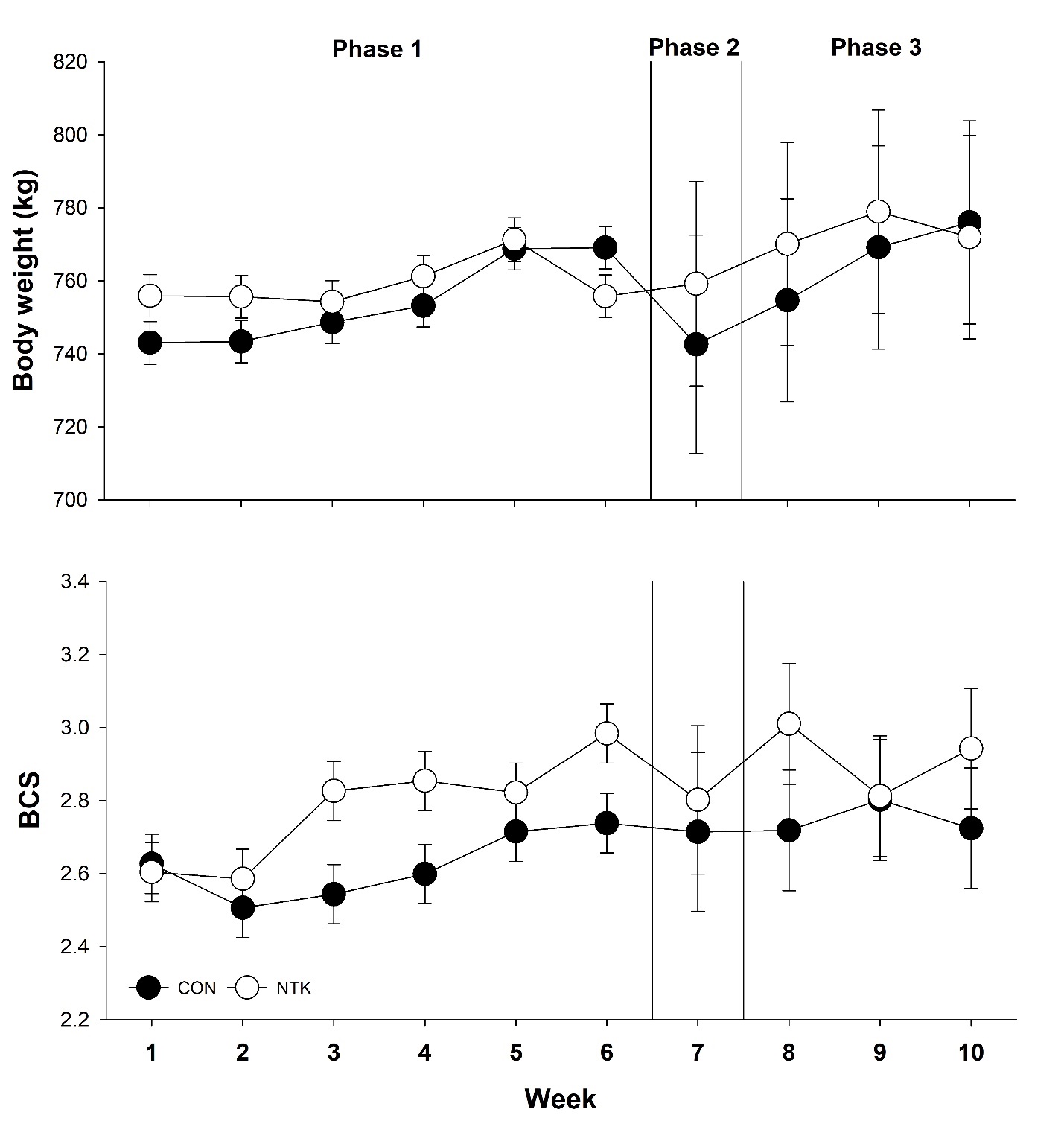


**Supplemental Figure 3.** Milk composition of cows supplemented with a *Saccharomyces cerevisiae* fermentation product (NTK) or fed a control diet (CON) subjected to an intramammary inflammation challenge with *Streptococcus uberis*. Data are separated according to experimental period: treatment feeding (Phase 1; 0-44 d), bacteria inoculation and challenge (Phase 2; 45-53 d), post antibiotic recovery (Phase 3; 54-75 d). No data were collected for Phase 2 due to blood contamination from the biopsy procedure performed on day 46.


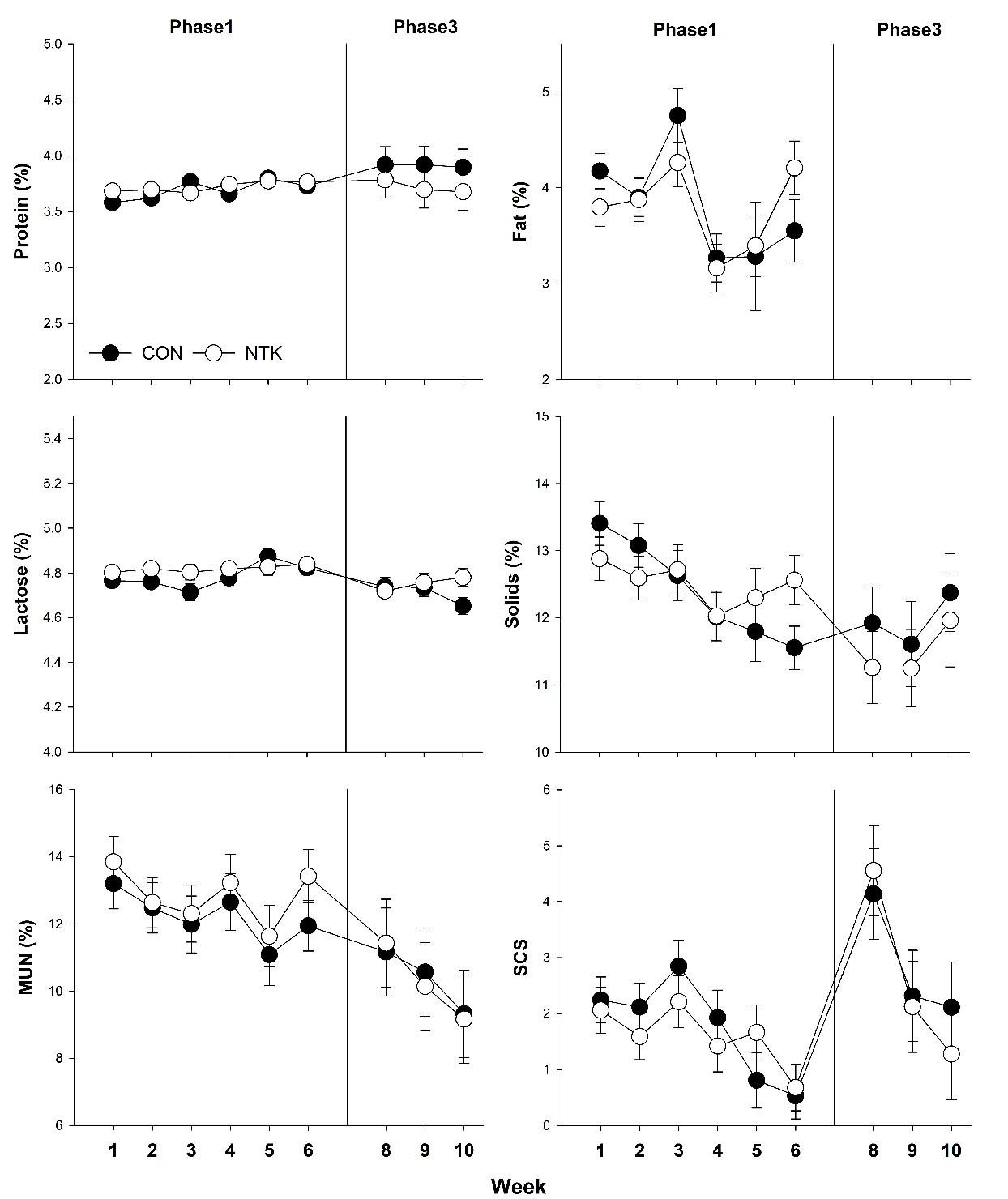


**Supplemental Figure 4.** Blood biomarkers of metabolic status in cows supplemented with a *Saccharomyces cerevisiae* fermentation product (NTK) or fed a control diet (CON) subjected to an intramammary inflammation challenge with *Streptococcus uberis* after 45 d of supplementation.


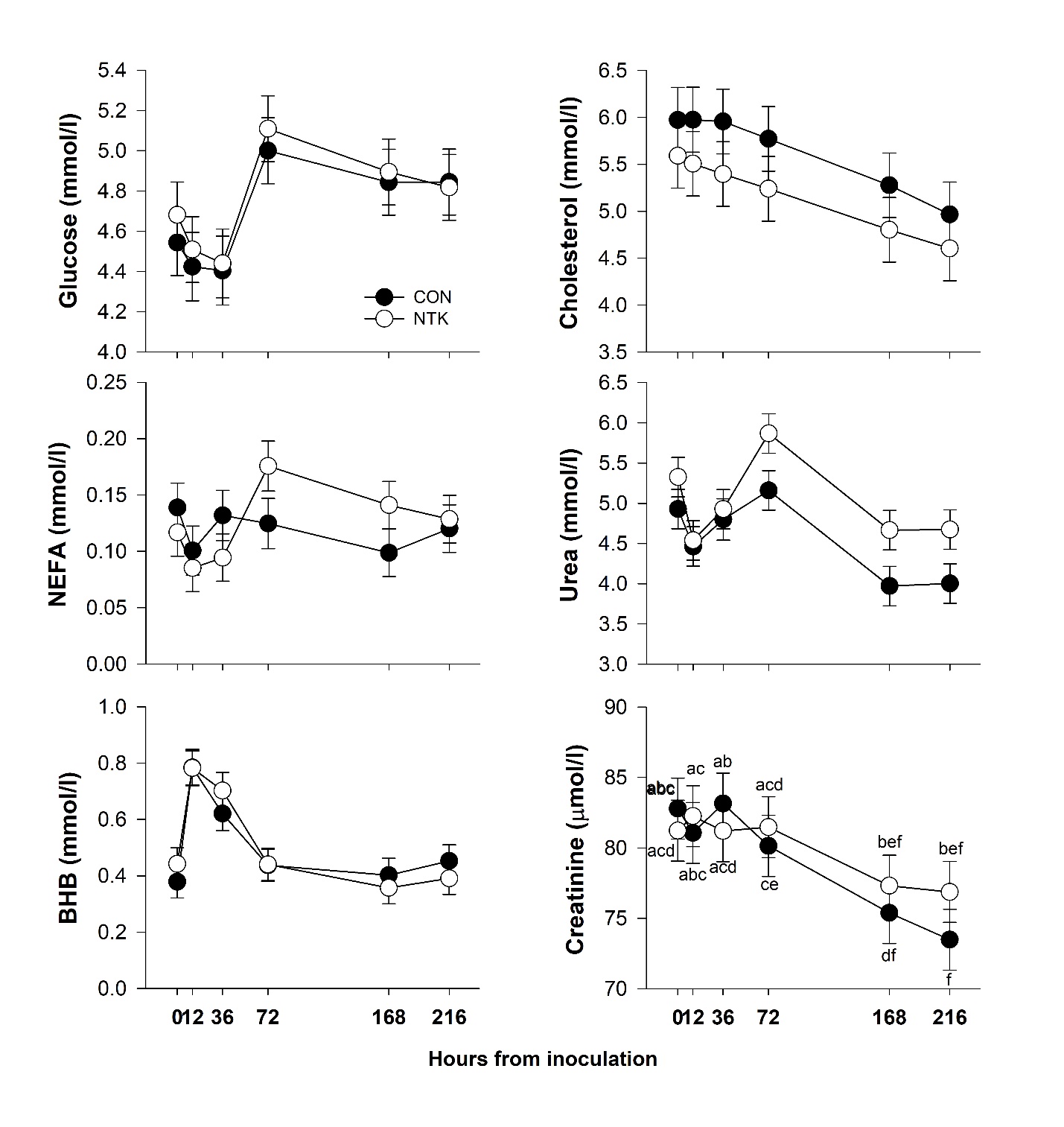


**Supplemental Figure 5.** Blood biomarkers of inflammation and acute phase response in cows supplemented with a *Saccharomyces cerevisiae* fermentation product (NTK) or fed a control diet (CON) subjected to an intramammary inflammation challenge with *Streptococcus uberis* after 45 d of supplementation. Different superscripts indicate significant difference (*P* < 0.05) between values and are reported when the interaction of treatment and time is significant (*P* < 0.05).


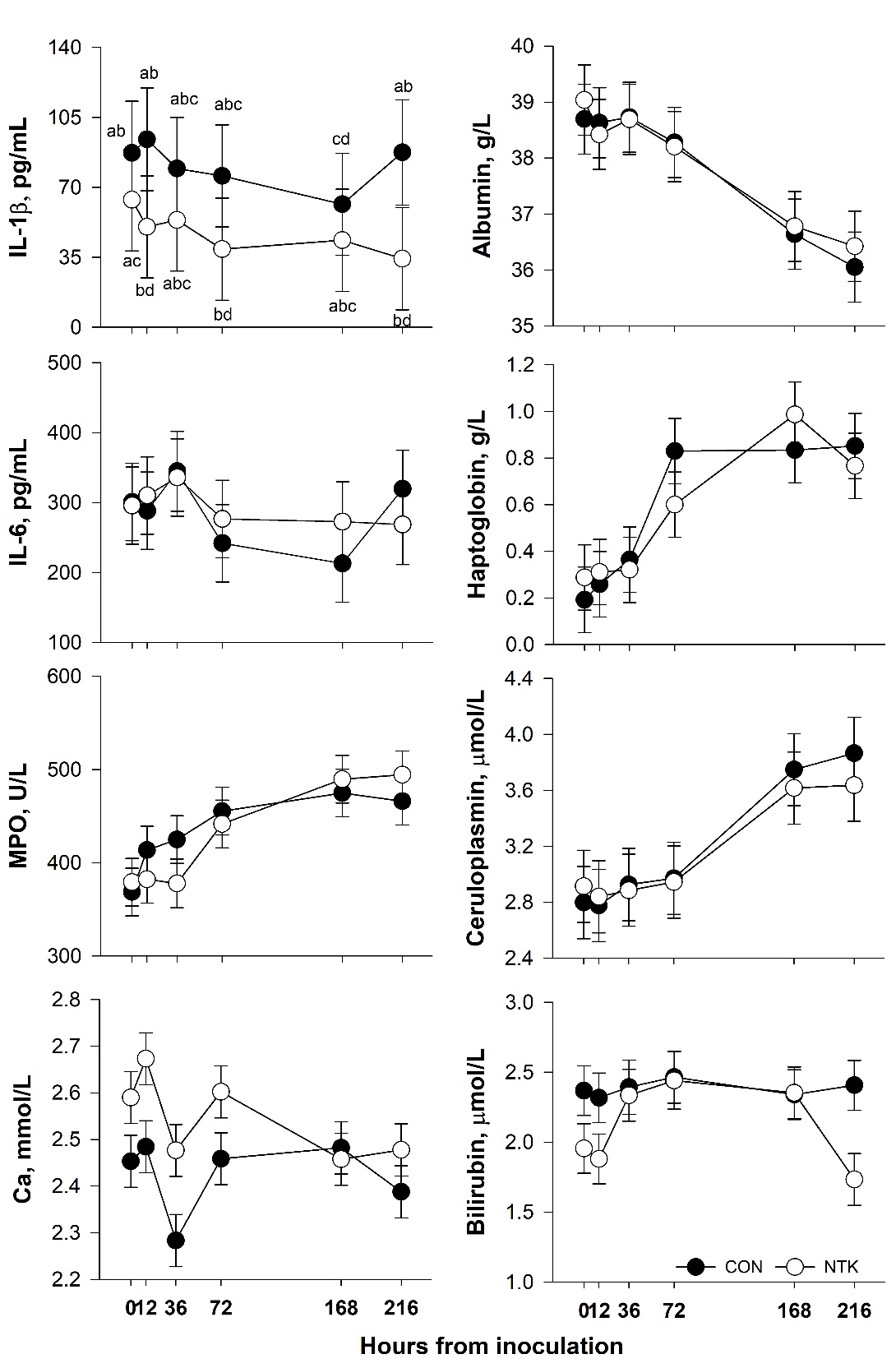


**Supplemental Figure 6.** Blood biomarkers of oxidative status in cows supplemented with a *Saccharomyces cerevisiae* fermentation product (NTK) or fed a control diet (CON) subjected to an intramammary inflammation challenge with *Streptococcus uberis* after 45 d of supplementation. Different superscripts indicate significant difference (*P* < 0.05) between values and are reported when the interaction of treatment and time is significant (*P* < 0.05).


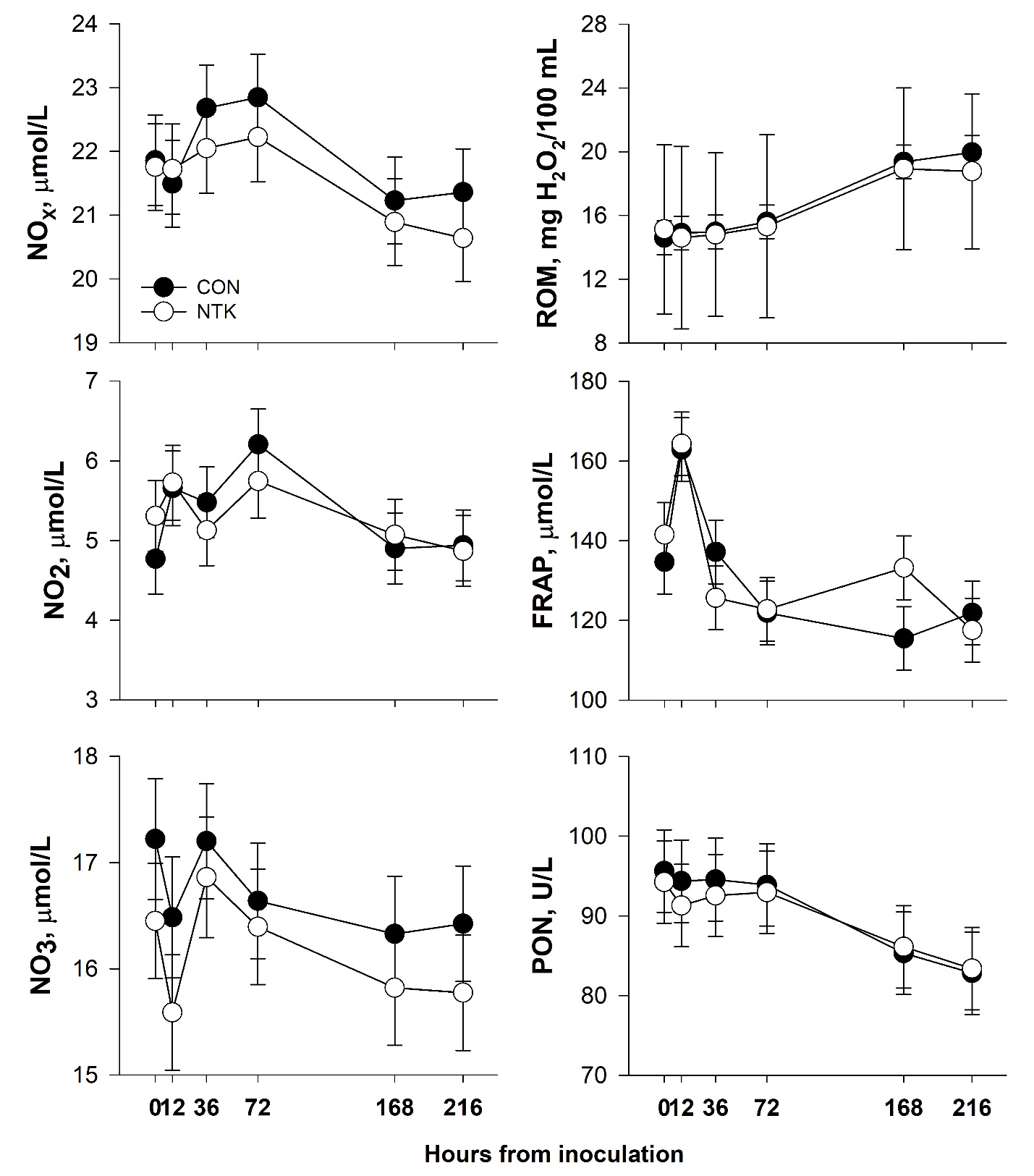


**Supplemental Figure 7.** Blood concentration of liver enzymes in cows supplemented with a *Saccharomyces cerevisiae* fermentation product (NTK) or fed a control diet (CON) subjected to an intramammary inflammation challenge with *Streptococcus uberis* after 45 d of supplementation.


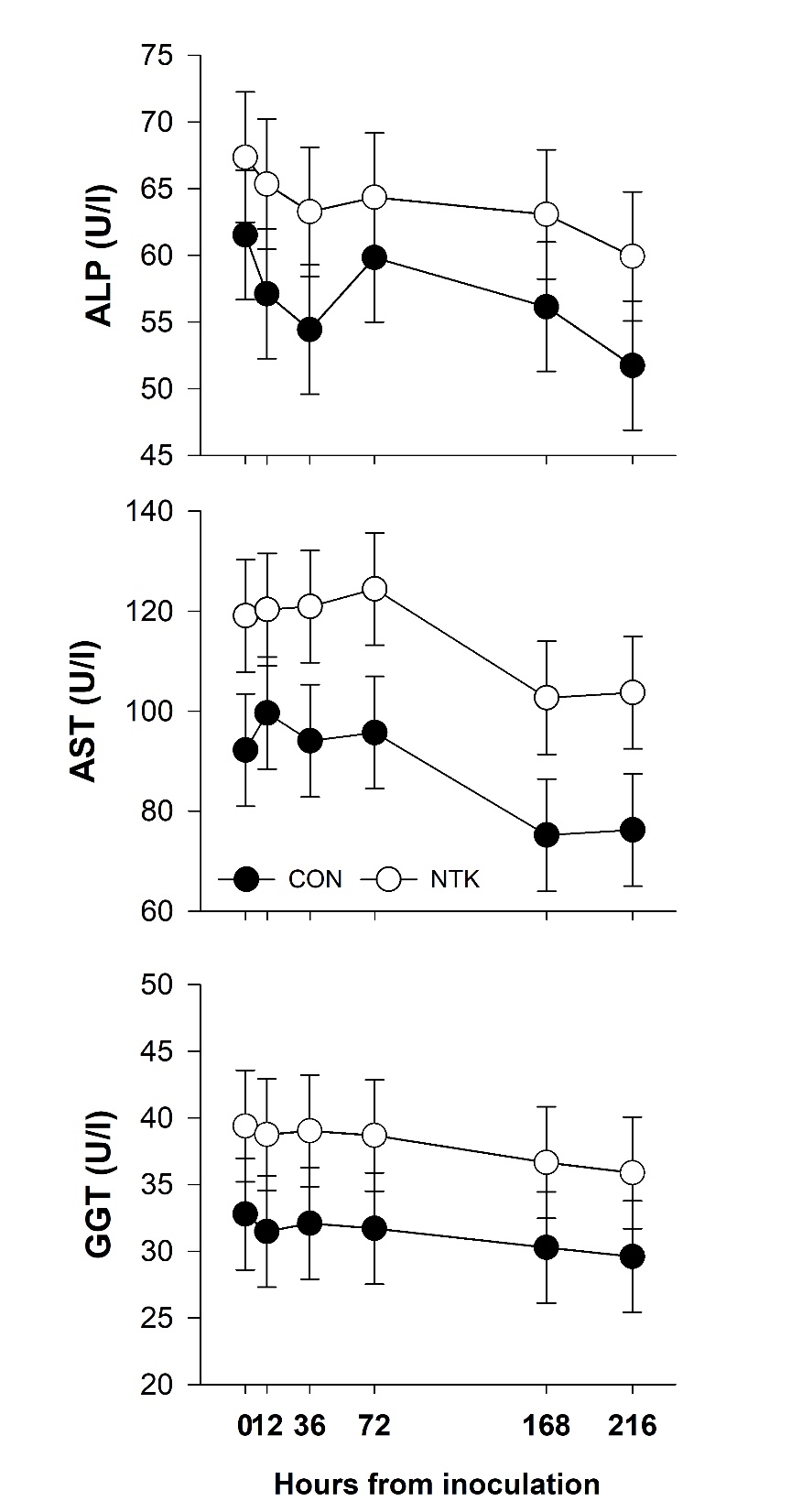


**Supplemental Figure 8.** Blood minerals concentration in cows supplemented with a *Saccharomyces cerevisiae* fermentation product (NTK) or fed a control diet (CON) subjected to an intramammary inflammation challenge with *Streptococcus uberis* after 45 d of supplementation.


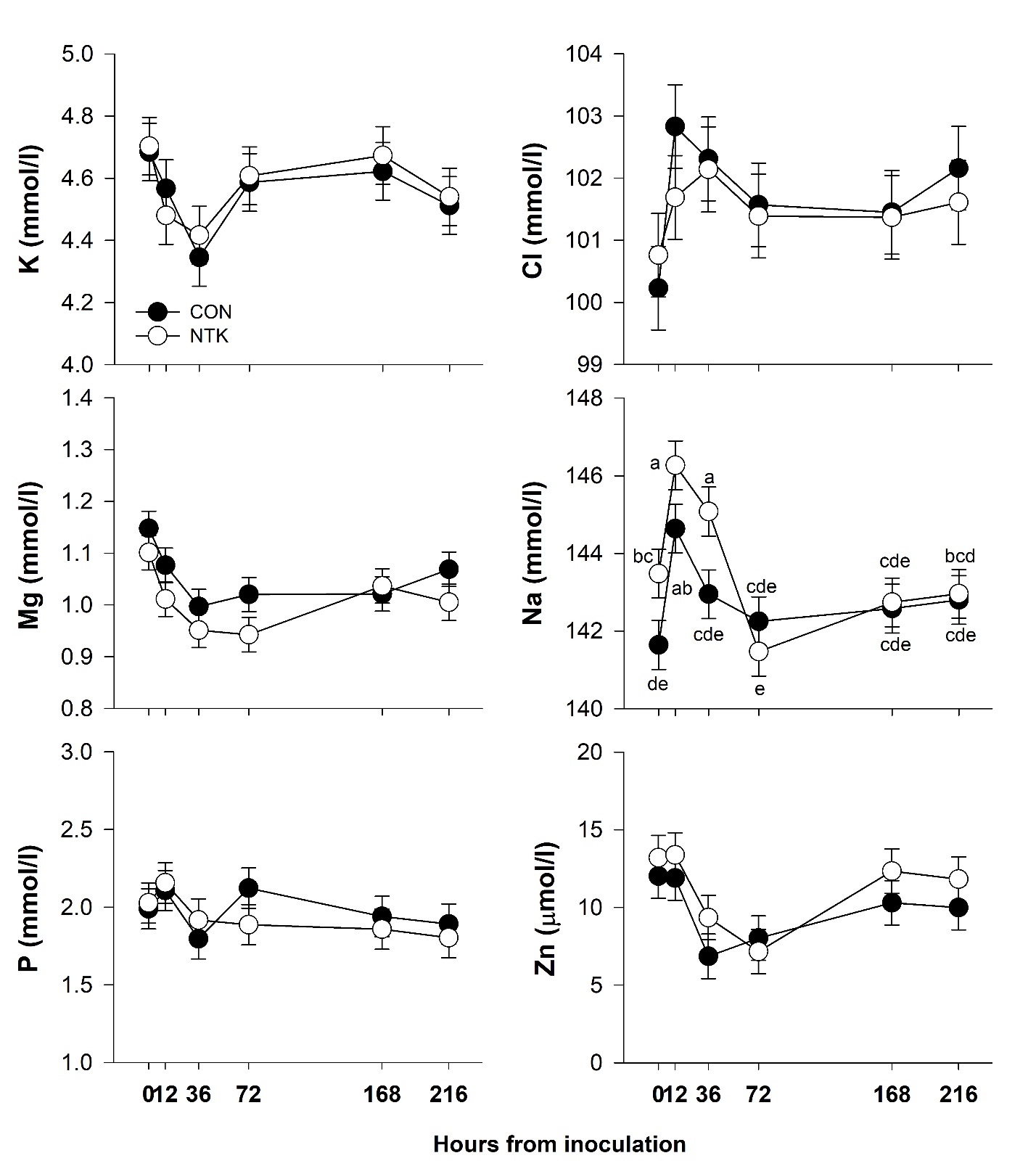


**Supplementary Figure 9.** Summary of the Dynamic impact analysis conducted on differentially expressed genes in liver and mammary gland tissue collected 36 h post mammary gland inoculation with *Streptococcus uberis* in dairy cows supplemented with a *Saccharomyces cerevisiae* fermentation product (NTK) compared to animals fed a control diet (CON). The impact (blue) represents the biological importance of the pathway, while the impact its direction of regulation. Positive flux (red) indicate upregulation, while negative flux (green) indicates downregulation of a pathway activity in the comparison NTK vs. CON.


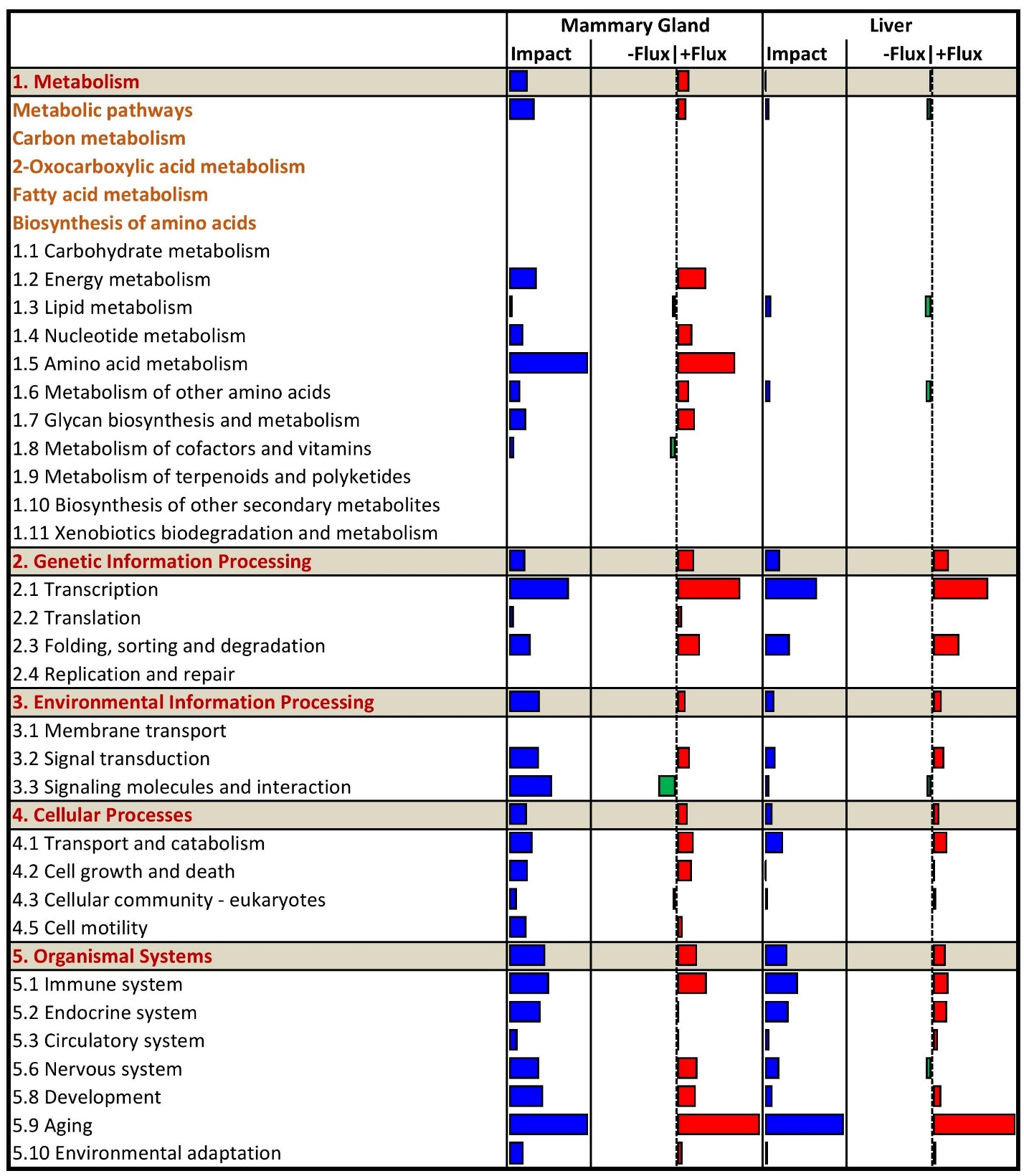


**Supplemental Figure 10.** Activity of pathway in liver tissue collected 36 h post mammary gland inoculation with *Streptococcus uberis* in dairy cows supplemented with a *Saccharomyces cerevisiae* fermentation product (NTK) compared to animals fed a control diet (CON). The impact (blue) represent the biological importance of the pathway, while the impact its direction of regulation. Positive flux (red) indicate upregulation, while negative flux (green) indicate downregulation of a pathway activity in the comparison NTK vs. CON.


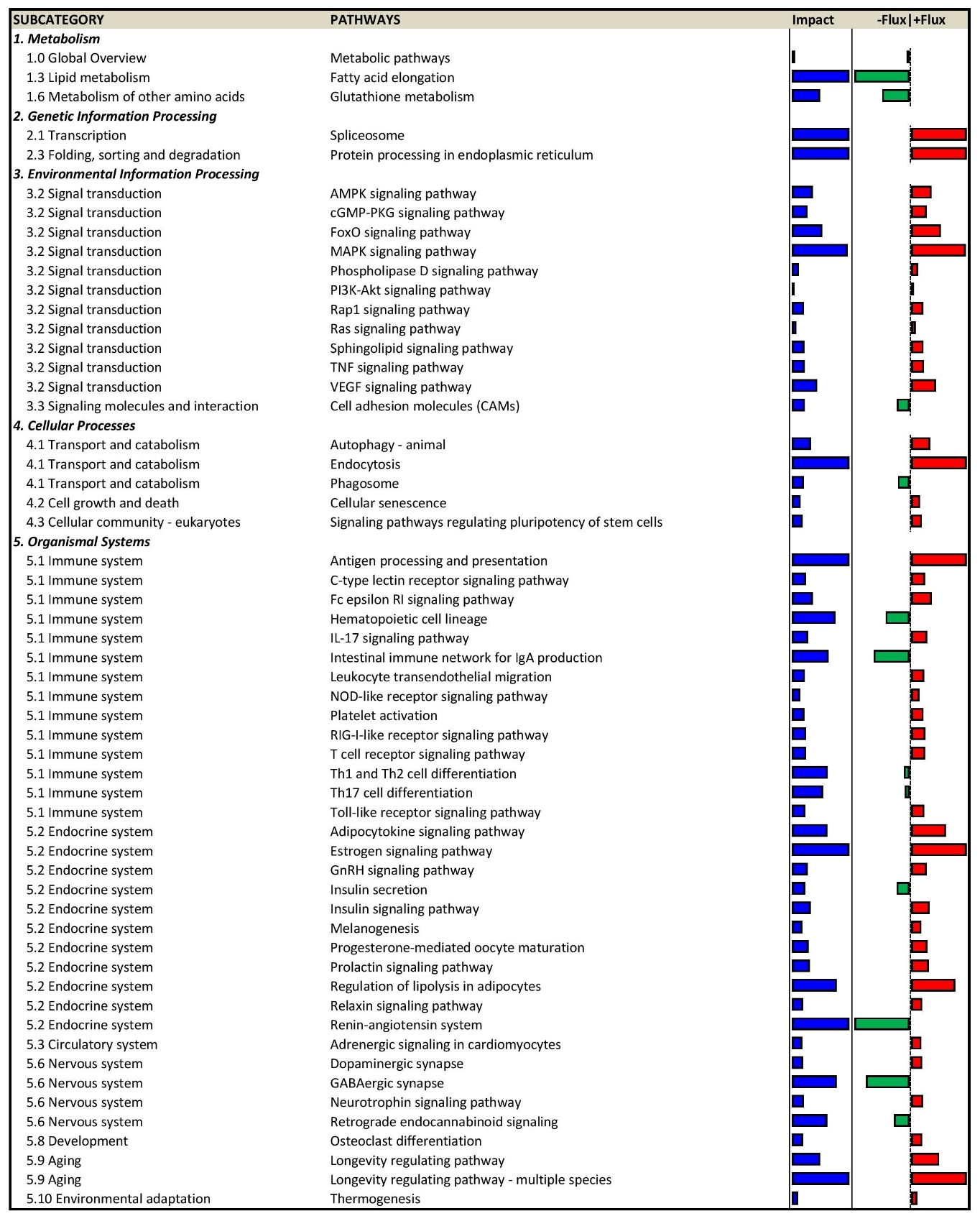

Supplement: Supplementary file 1 — Additional file 1. Provides detailed about diet composition (Suppl. Table 1), animal data prior to experiment (Suppl. Table 2), and complete results about the RNA-sequencing analysis, including sequencing and alignment performance (Suppl. Table 3), and full list of differentially expressed genes with relative fold-changes (Suppl. Tables 4, 5, and 6). Furthermore, it includes graphical representation of results reported only in tabular format in the main body: DMI as %BW (Suppl. Fig. 1), BW and BCS (Suppl. Fig. 2), milk composition (Suppl. Fig. 3), blood biomarkers of metabolic status, inflammation and APP, oxidative status, liver enzymes, and minerals (Suppl. Figs. 4, 5, 6, 7, and 8). Included are also the full results of liver DIA pathway analysis (Suppl. Fig. 10). [file 40104_2021_560_MOESM1_ESM.docx]
